# Cross sectional study on the competence and confidence of dental students and graduates in the management of medically compromised patients and acute medical emergencies

**DOI:** 10.1371/journal.pone.0281801

**Published:** 2023-02-15

**Authors:** Timothy Jie Han Sng, Chee Weng Yong, Raymond Chung Wen Wong

**Affiliations:** Faculty of Dentistry, National University of Singapore, Singapore, Singapore; Shahid Beheshti University of Medical Sciences, School of Dentistry, ISLAMIC REPUBLIC OF IRAN

## Abstract

A rapidly aging population means many people have multiple health issues leading to an increased risk of acute medical emergencies. The objective of this study was to evaluate how essential experiential learning is in developing dental graduates’ ability to manage medically compromised patients. Three hundred and twenty-seven students and graduates were invited to participate in an online survey to rate their confidence in managing medically compromised patients and acute medical emergencies using a 5-point Likert scale. Competence of knowledge was evaluated using 30 multiple choice questions (MCQs) across six domains. The respondents were also asked whether a theory-only training adequately prepared them to manage medically compromised patients, or whether it must be supplemented with clinical training. Two-hundred and sixty-four responses were collected from 75 undergraduates (UG), 96 junior dental officers (JDO) and 93 senior dental officers (SDO). The UG reported that they infrequently managed medically compromised patients, whereas both the JDO and SDO reported having frequent encounters with these patients. The mean confidence scale in the management of medically compromised patients were 2.62, 3.50 and 3.69 (out of 5), respectively. In contrast, their confidence scale in the management of acute medical emergencies was 2.05, 2.33 and 2.50 (out of 5), respectively. The MCQ scores were 25.51, 26.44 and 26.86 out of 30, respectively. The outcomes of the JDO and SDO were significantly better than the UG (t-tests, p<0.05). All three groups responded that a theory-only training in dental school did not adequately prepare them to manage medically compromised patients. Both the JDO and SDO felt that their clinical work experience better prepared them to manage these patients. Experiential learning from “real-life” clinical experience is an essential component in developing graduates’ confidence and competence in the management of medically compromised patients. A dental curriculum with theory-only training in this aspect is inadequate.

## Introduction

Experiential learning is defined as students’ construction of knowledge and meaning through active participation in a “real-life” experience [1]. Yardley described that in the context of medical/dental education, the term is applied to experiences which have been included in the curriculum to bring the student into contact with others in a particular role and context [1]. In the context of dentistry, it would mean bringing dental students out of the classroom and allowing them to participate in direct patient care. This concept is not foreign to most dental schools since dental students are typically allowed to manage and treat patients under staff supervision [2, 3].

The objective of the dental school curriculum is to equip graduates with the necessary skills and knowledge to embark on their careers. In a span of a few years, students are required to acquire a significant amount of information and to learn a variety of dental procedures [4]. Due to limited time, the school must select what is necessary for graduates to practice dentistry safely. As such, certain skills or knowledge that are deemed too advanced at the undergraduate level may not be prioritized [5].

An example would be the management of medically compromised patients and acute medical emergencies [5–7]. Compared to the typical patient, a medically compromised patient often requires special considerations in terms of patient care and treatment planning. For example, a patient on anti-coagulant is at high risk of excessive bleeding and precautions must be taken to prevent this. When treating medically compromised patients, the dentist must be able to cater their treatment according to the patient’s health state and provide first-line care for medical emergencies in the dental clinic setting [8]. Some examples of acute medical emergencies that may occur during dental treatment include cardiac arrest, asthma attack and seizures. Unfortunately, there are no existing data in Singapore that reports on the incidence of acute medical emergencies in dental clinics, and we can only rely on inaccurate anecdotal experience and recounting of incidences among colleagues. Inadequate assessment of a patient’s susceptibility to such emergencies and mishandling of these emergencies can lead to dire consequences [9, 10]. Having considered a patient’s medical and dental conditions, a dentist must then determine the urgency and necessity of a dental treatment based on a risk-benefit analysis. If necessary, the dentist must defer the appointment until the patient’s medical condition is better optimized for dental procedures [11]. For instance, patients who had a recent acute myocardial infarction should have a non-urgent elective dental treatment postponed until a few weeks later.

Medically compromised patients require more than routine delivery of care, so it is not surprising that many dental schools omit clinical training for this subgroup of patients [12]. This would mean that dental graduates may only have a theoretical understanding of the management of these patients, without any experiential learning. While global health has improved over the years, a large fraction of the burden of diseases comes from non-communicable or chronic diseases such as cardiovascular disorders and stroke [13]. Thus, dental professionals can anticipate a greater proportion of medically compromised patients in their clinical practice. However, with solely theoretical education, graduates may not be adequately prepared to treat medically compromised patients [14, 15], or to handle acute medical emergencies in a dental setting [16–20]. This may not be ideal for patient safety.

Among all the subjects taught in dental schools, dental graduates from Hong Kong felt the least prepared in the management of patients with medically compromised conditions and acute medical emergencies [6]. The majority of dental students from the United States were not comfortable managing medically compromised geriatric patients [21]. Similar responses were provided by dental graduates [15]. Only 30% of general dental practitioners based in the United Kingdom felt adequately prepared to manage acute medical emergencies upon graduation [22]. More than half of the general dental practitioners surveyed in New Zealand responded that the training for acute medical emergencies during dental school were not satisfactory and 14.1% felt inadequate to manage these patients [16]. Similarly, more than half of the dentists surveyed in Germany were not confident in providing basic life support in a dental setting [23]. This was corroborated by similar studies conducted in Latin America [7].

This problem was also identified in Singapore. Regardless of dental needs, patients under the care of dental students are usually screened based on their medical history before even being considered suitable for undergraduate dental treatment. Medically compromised patients (for example, those on dual-anti platelet therapy, ongoing cancer treatment, etc.) would be referred instead to the hospital dental specialists or dental officers for dental management.

The objective of the present study was to evaluate whether experiential learning affects the confidence and competence of dental undergraduates (UG), Junior Dental Officers (JDO) and Senior Dental Officers (SDO) in the management of medically compromised patients and acute medical emergencies in a dental setting.

## Materials and methods

A descriptive cross-sectional study was conducted electronically via an online survey platform from April to June 2021. This study was approved by the National University of Singapore Institutional Review Board prior to commencement (Reference no. 2021–390). The need for consent was waived by the ethics committee because there was no personal data or identifiers collected.

Recent dental graduates (Graduated in the academic year 2017–2020) and undergraduates in their clinical years (3^rd^ and 4^th^(Final) year of program) from Singapore were recruited. The electronic survey was distributed via the respective cohort representatives. The respondents were stratified into three groups according to their experience level: UG–Respondents who were in the 3^rd^ or final year of dental school (cohort size = 107), JDO–Respondents who graduated in 2019 or 2020 (cohort size = 103) and SDO–Respondents who graduated in 2017 or 2018 (cohort size = 117). Based on the sample size calculations, 90, 82 and 84 respondents were required for the UG, JDO and SDO groups respectively (95% confidence interval, 5% margin of error).

The survey consisted of four sections. The first section collected the following demographic details from the respondents: Gender, Year of Graduation (2017, 2018, 2019 or 2020) or Year of study (3^rd^ Year or 4^th^/Final Year), Work experiences, and Exposure to medically compromised patients (Table 1).

**Table 1:** Demographic profile of respondents.

		UG		JDO		SDO	
**No. of respondents**		**75**		**96**		**93**	
**Year of study / graduation**		Year 3	43	2020	40	2018	56
		Year 4	32	2019	56	2017	43
**Gender**	**M**	30		39		31	
	**F**	45		57		62	
**Workplace**	**Public Service**	0		94		73	
	**Military Practice**	0		1		3	
	**Private Practice**	0		1		17	
**Worked in SOC/MSS**		N/A		17		79	
**Exposure**	**Never**	15		4		0	
	**Infrequently**	58		27		17	
	**Frequently**	2		44		55	
	**Every working Day**	0		21		21	

Abbreviations:

Exposure = Exposure to medically compromised patients; UG–Undergraduate; JDO–Junior dental officer; SDO–Senior dental officer, SOC/MSS–Specialist outpatient clinics/Multi-specialist service

The second and third section sought to assess the respondents’ confidence and competence in the management of medically compromised patients. It consisted of six domains: cardiovascular disorders, respiratory disorders, neurological disorders, hematological disorders, endocrine disorders and malignancy. The six chosen domains were important to clinical dentistry as these conditions are more likely to affect treatment decision and are relatively more prevalent [24].

For the second section, the respondents were required to indicate their level of confidence in managing the patients and also, in providing management for acute medical emergencies of each domain. This was assessed on a 5-point Likert scale whereby the respondents were to rate their confidence level from 1 to 5 (1- Not confident 2- Slightly confident, 3- Somewhat confident, 4- Fairly Confident and 5- Very confident). The total scores within each domain and across all the domains were calculated.

For the third section, respondents were asked to answer five multiple-choice questions (MCQs) in each of the six medical domains to assess their knowledge (Table 2). For each MCQ, only 1 out of the 4 options was the correct answer. MCQ was selected as an assessment tool due to its objectivity and reliability [25]. Compared to direct workplace observation or clinical simulations, MCQs were also a more feasible and standardized form of assessment in the context of our study. Lastly, MCQs served as a valid measurement of clinical competence, namely that of basic facts and applied knowledge [25, 26]. The MCQs were vetted by our co-author RCW who was the NUS undergraduate oral and maxillofacial surgery (OMS) module coordinator for management of medical emergencies and medically compromised patients from 2013 to 2021. Similarly, the total scores within each domain and across all the domains were calculated.

**Table 2:** Questionnaire on the six domains and respondents’ answers.

		UG	JDO	SDO
**Domain 1: Cardiovascular**				
Q1.1. At what values of blood pressure should elective treatment be deferred and resumed after it is controlled?	(A) 140/90	3	2	2
	(B) 160/100	26	29	14
	(C) 180/100	40	50	67
	(D) All of the above	6	15	10
Q1.2. Which of the following is NOT a valid consideration for patients with hypertension?	(A) Prophylactic doubling of anti-hypertensive medication dosage in the morning	57	84	80
	(B) Limit to a maximum of 2 cartridges of local anesthetic with 1:100000 epinephrine	11	8	7
	(C) Avoid erythromycin or clarithromycin in patients who are taking calcium channel blockers as it can lead to acute renal failure	7	1	6
	(D) Aim for short, pain-free, stress-free appointments	0	3	0
Q1.3. Which of the following is NOT a possible sign of myocardial infarction?	(A) Dyspnea	9	2	5
	(B) Diaphoresis	3	8	6
	(C) Chest pain	0	0	0
	(D) Hemopytsis	63	86	82
Q1.4. How long should dentists wait before performing elective treatment on a patient with myocardial infarction?	(A) 2 weeks for non-invasive treatment, 2 months for complex invasive procedure(A) 2 weeks for non-invasive treatment, 2 months for complex invasive procedure	0	0	0
	(B) 2 weeks for non-invasive treatment, 6 months for complex invasive procedure	7	4	3
	(C) 6 weeks for non-invasive treatment, 2 months for complex invasive procedure	0	2	3
	(D) 6 weeks for non-invasive treatment, 6 months for complex invasive procedure	68	90	87
Q1.5. Which of the following do NOT require antibiotic prophylaxis to prevent infective endocarditis?	(A) Prosthetic cardiac valve	4	1	0
	(B) Previous infective endocarditis	0	1	1
	(C) Coronary artery bypass grafting	71	94	92
	(D) Unrepaired cyanotic congenital heart disease	0	0	0
**Domain 2: Respiratory Disorders**				
Q2.1. Which of the following advice for asthmatic patient is NOT appropriate?	(A) Advise late morning or afternoon appointments as respiratory symptoms are worse in the early morning	1	6	8
	(B) Advise patient to bring his or her inhaler for each appointment	1	1	0
	(C) Advise patient to discontinue any steroid medications for at least 1 week before any surgical procedure	72	89	90
	(D) All of the above	1	0	2
Q2.2. In the event of asthmatic attack, which of the following should be done?	(A) Stop work immediately and put patient upright	0	0	0
	(B) Give the patient his or her usual inhaled bronchodilator	2	6	1
	(C) Consider nebulized salbutamol, supplementary oxygen or even subcutaneous epinephrine	1	0	1
	(D) All of the above	72	90	91
Q2.3. Which of the following is NOT a sign of a poorly controlled chronic obstructive pulmonary disease?	(A) Dyspnea	0	0	0
	(B) Oxygen saturation less than 96%	0	0	0
	(C) Breathlessness at rest	0	1	1
	(D) Persistent high fever	75	95	92
Q2.4. Which of the following should be done if you suspect that the patient has aspirated a foreign body?	(A) If the patient is asymptomatic, there is no need to refer the patient for further management in the hospital	8	1	2
	(B) Quickly put the suction into the patient’s throat to suck out the foreign body	6	8	6
	(C) Put the patient in reverse Trendelenburg position and encouraged coughing	49	63	73
	(D) Ask the patient to check his or her stool to ensure that the foreign body has been passed out	12	24	12
Q2.5. Which is the following is NOT an appropriate precaution when prescribing medications to patient with respiratory disorders?	(A) Avoid aspirin as it can precipitate an asthmatic episode	3	8	4
	(B) Avoid barbituates as analgesics as it can result in respiratory depression	7	3	3
	(C) Avoid macrolides if the patient is on theophylline as it can lead to a series of side effects such as anorexia. Insomnia etc	6	4	11
	(D) Avoid anti-histamines as it can lead to severe headache	59	81	75
**Domain 3: Neurological Disorders**				
Q3.1. Which of the following is a sign of a poorly controlled epileptic patient?	(A) More than 1 seizure per month	75	94	90
	(B) 1 seizure episode in the past year	0	0	0
	(C) Gingival overgrowth	0	0	1
	(D) Trembling of the hands	0	2	2
Q3.2. Which of the following is/are considerations for patient with dementia?	(A) Lack of decision making capacity	4	3	5
	(B) Patient’s movements must be watched as they are prone to falls	0	0	0
	(C) They may have difficulties maintaining their oral hygiene	0	0	1
	(D) All of the above	71	93	87
Q3.3. In a Grand Mal episode, which of the following should NOT be done?	(A) Rule out other potential mimics such as syncope, myocardial infarction, hypoglycemia	0	3	4
	(B) Stop all work, remove all instruments, debris, secretions and fluid from mouth	0	0	3
	(C) Put a mouth block or prop to prevent the patient from biting his or her tongue	75	83	85
	(D) Put dental chair in a supine position and bring it as close to the ground as possible	0	10	1
Q3.4. Which of the following is NOT a potential oral side effects of taking anti-convulsant?	(A) Gingival hyperplasia	1	3	0
	(B) Tongue Spasms	67	84	74
	(C) Dysgeusia	3	5	18
	(D) Stomatitis	4	4	1
Q3.5. Which of the following is NOT an appropriate precaution when managing patient with autism spectrum disorder?	(A) Ask parents or caregiver what makes the child comfortable and uncomfortable	0	0	0
	(B) Make use of social stories to prepare patients for their first dental appointment	0	0	1
	(C) Antibiotic prophylaxis to prevent infection	75	95	92
	(D) Ask a legal care-giver to accompany if patient is deemed to be unable to give consent	0	1	0
**Domain 4: Bleeding Disorders**				
Q4.1. Which of the following is true?	(A) All patients on warfarin must bridge to heparin prior to any invasive procedures	0	1	0
	(B) All patients on single anti-platelet therapy must stop their anti-platelet medications 3 days before extraction	3	1	0
	(C) All patients on warfarin should have their INR checked prior to invasive dental procedures	71	92	91
	(D) All of the above	1	2	2
Q4.2. What is the recommended minimum platelet count for performing routine dental procedures (SAP & Xn)	(A) <30,000	8	2	4
	(B) 30,000–50,000	26	30	38
	(C) >50,000	38	53	35
	(D) >80,000	3	9	16
Q4.3. Which of the following is NOT an appropriate measure to reduce post-operative bleeding?	(A) Adequate analgesics	0	0	0
	(B) Tranexamic acid mouth washes	73	94	92
	(C) Surgicel application	1	1	1
	(D) Pressure haemostasis with gauze	1	1	0
Q4.4. Which is of the following is NOT a valid consideration for patients with haemophilia	(A) Advise appointments (especially surgical procedures) to be at the beginning of the day	44	21	27
	(B) Prolonged observation period to ensure haemostasis	0	3	2
	(C) INR must be checked prior to treatment	29	67	63
	(D) Atraumatic extraction to minimize bleeding	2	5	1
Q4.5. Which of the following patients may have bleeding risks?	(A) Patient on aspirin	8	0	0
	(B) Patient on rivaroxaban	0	0	1
	(C) Patient on warfarin	1	0	0
	(D) All of the above	66	96	92
**Domain 5: Endocrine Disorders**				
Q5.1. Which of the following is true about patients with uncontrolled diabetes?	(A) They are at risk of infection as due to altered immune function	4	1	4
	(B) They are at risk of hyperglycemic crisis	0	0	0
	(C) They are at risk of diabetic ketoacidosis	1	1	2
	(D) All of the above	70	94	87
Q5.2. Which of the following is/are appropriate precaution(s) for patient with adrenal insufficiency?	(A) Advise patients to have their appointments first thing in the morning when cortisol levels are the highest	2	8	4
	(B) Watch out for hypotension or hypoglycemia during the procedure	19	11	7
	(C) Consider prophylactic steroids if patient is on systemic corticosteroids for the past 2–4 weeks	2	7	5
	(D) All of the above	52	70	77
Q5.3. In the event of thyroid storm, which of the following should be performed?	(A) Terminate all dental procedures	2	5	2
	(B) Put patient on supine position with legs elevated slightly	0	1	1
	(C) Provide basic life support until the arrival of emergency medical services	4	0	0
	(D) All of the above	69	90	90
Q5.4. Which is of the following is NOT a valid consideration for pregnant patients	(A) Possibility of supine hypotension syndrome in 3^rd^ trimester	15	10	3
	(B) Avoid radiography as much as possible	1	0	1
	(C) Mepivacaine should be used instead of lidocaine as the latter is a Class D drug.	59	84	89
	(D) Avoid medications which can have effects on developing fetus	0	2	0
Q5.5. Which of the following is false?	(A) Patients on insulin may be at risk of hypoglycemia	0	0	0
	(B) Avoid Nyastatin mouthwashes in diabetic patients as it contains sugar which may affect their blood glucose control	68	87	82
	(C) Corticosteroids may affect the blood glucose control of diabetic patients	2	8	8
	(D) Diabetic patients with sweet smelling breath may be suspicious of ketoacidosis	5	1	3
**Domain 6: Malignancy**				
Q6.1. What of the following therapies can benefit from dental clearance / examination to prevent complications?	(A) Radiotherapy	12	11	4
	(B) Chemotherapy	0	1	0
	(C) Haemotopoetic stem cell transplant	0	0	1
	(D) All of the above	63	84	88
Q6.2. Which of the following should be advised to patients who had radiotherapy to the head and neck region?	(A) Maintain oral hygiene to prevent caries as they may have hyposalivation	2	0	0
	(B) Jaw opening exercise to reduce restrictions in mouth opening as a result of fibrosis	0	1	0
	(C) Avoid dental extractions and consider other alternatives such as root canal therapy to prevent osteonecrosis of the jaw	5	0	1
	(D) All of the above	68	95	92
Q6.3. Which of the follow is NOT an appropriate consideration for patient on chemotherapy?	(A) They may have increased bleeding risk	7	9	7
	(B) They may be anaemic	1	0	1
	(C) Emergency dental treatment can be done only after all chemotherapy cycles have been completed	65	83	85
	(D) They have increased risk of infection	2	4	0
Q6.4. Which is of the following is NOT a criterion for the diagnosis of a medication related osteonecrosis of the jaw?	(A) Current or previous treatment with anti-resorptive or anti-angiogenic agent	0	0	2
	(B) Pus formation from an exposed bone	57	55	56
	(C) Exposed bone or bone that can probed through a fistula in the maxillofacial region that has persisted for more than 8 weeks	2	2	1
	(D) No history of radiotherapy to the jaws or obvious metastatic disease to the jaws	16	39	34
Q6.5. Which of the following is NOT associated with medication related osteonecrosis of the jaw?	(A) Simvastatin	66	94	92
	(B) Zoledronate	8	1	0
	(C) Denosumab	0	0	1
	(D) Bevacizumab	1	1	0

Abbreviations:

SAP & Xn–Scaling and polishing & Simple extractions; UG–Undergraduate; JDO–Junior dental officer; SDO–Senior dental officer.

In the fourth section, the respondents were asked whether they perceived the theory-only training provided by dental school to be sufficient and if experiential learning from clinical practice was necessary to prepare them for the management of medically compromised patients. The theory-training that the respondents received included four hours of lectures and tutorials for managing acute medical emergencies and medically compromised patients. This didactic content was supplemented with content which covered oral medicine and internal medicine knowledge. The learning objectives for these two topics are listed in Table 3.

**Table 3:** Learning objectives by topics in the course.

Topics	Learning Objectives
**Medically Complex patients**	Familiar with the management of patients with common diseases like diabetes mellitus, hypertension, steroid therapy, anticoagulants, valve replacement Able to describe the basic principles of management of the above diseases and common medications given Able to describe complications that may arise during treatment
**Medical Emergencies in Dentistry**	Familiar with the management of common medical emergencies Able to describe the layout of the emergency trolley, its uses and how to protect an airway Familiar with the principles of basic life support

### Statistical analysis

Quantitative analysis was performed using IBM SPSS (Version 26.0. Armonk, NY: IBM Corp). The normality of the results were assessed using Shapiro-Wilk test. T-tests was performed to compare the discrete variables between the groups whereas, Chi-square test was done to evaluate the differences in categorical variables between the groups. Statistical significance level was set at a p-value of <0.05.

## Results

### Section 1: Demographic profile

There was a total of 268 responses to the survey. This meant that 81.9% of the population size responded to this survey. All respondents were from the only dental school in Singapore. Four responses were excluded as they were incomplete. Hence, a total of 264 responses were included in the analysis.

The number of respondents from each group were as such:

1. UG–Respondents who were in the 3^rd^ or 4^th^ year of dental school (n = 75);
2. JDO–Respondents who graduated in 2019 or 2020 (n = 96);
3. SDO–Respondents who graduated in 2017 or 2018 (n = 93).

One hundred (37.9%) and 164 (62.1%) respondents were male and female, respectively. Most of the JDO and SDO who responded to the survey were in the public service (97.9% and 78.5%, respectively) Seventy-nine of the SDO (85%) had worked or were working in dental specialist center or in a multi-specialty service, whereas only 17 JDO (17.7%) had the same experience. The differences in the workplaces of the respondents were statistically significant (Chi-square, p<0.00001).

Exposure of the respondents to medically compromised patients were categorized into “never”, “infrequently”, “frequently” and “every working day”. The UG group mostly felt that they infrequently encountered such patients, while the JDO and SDO groups responded that they managed these patients frequently. The difference in the exposure of JDO and SDO managing these patients was significantly higher as compared to the UG (t-tests, p<0.00001 and p<0.00001, respectively). However, there were no statistically significant differences in the exposure of managing medically compromised patients between the JDO and SDO (p = 0.15). The summary of the demographic profiles of the respondents is shown in Table 1.

### Section 2: Confidence of the respondents

In contrast to both the JDO and SDO, the UG were less confident in the management of dental patients with stable medical conditions (Confidence scores of 15.76, 21.02 and 22.12, respectively), or in the management of unexpected acute medical emergency (Confidence scores of 12.27, 13.94. 15.00, respectively, Table 4). The mean score was determined by dividing the total score by six. Based on this, the mean score across the six domains would be 2.62, 3.50, 3.69, respectively. The average total score for the self-rated confidence in the management of acute medical emergencies in a dental setting for the UG, JDO, and SDO were 12.27, 13.94 and 15.00, respectively (mean of 2.05, 2.33, 2.50, respectively, across the six domains).

**Table 4:** Self-rated perceived confidence and MCQ score.

Domain / Score		Confidence in managing dental patients with stable medical conditions for each domain			Confidence in providing acute management of an unexpected medical emergency event for each domain			MCQ score achieved in each domain		
		UG	JDO	SDO	UG	JDO	SDO	UG	JDO	SDO
**Cardiovascular disorders**	**1**	9	0	0	27	39	30	1	1	0
	**2**	14	3	0	31	33	35	8	1	0
	**3**	32	16	14	6	15	23	11	5	3
	**4**	20	67	48	11	9	3	26	51	48
	**5**	0	10	31	0	0	2	29	38	42
**Respiratory disorders**	**1**	7	0	0	17	14	21	0	1	1
	**2**	24	3	5	29	38	32	1	2	0
	**3**	19	26	23	25	36	28	7	13	10
	**4**	24	53	47	4	7	7	31	26	27
	**5**	1	14	18	0	1	5	36	54	55
**Neurological disorders**	**1**	21	2	0	32	23	28	0	0	0
	**2**	18	12	17	23	38	35	0	1	1
	**3**	18	42	34	15	26	23	1	6	4
	**4**	18	35	33	5	7	4	10	15	26
	**5**	0	5	9	0	2	3	64	74	62
**Bleeding disorders**	**1**	9	0	0	12	1	0	0	1	0
	**2**	23	3	4	21	13	4	12	2	3
	**3**	20	36	20	38	41	20	21	19	14
	**4**	22	49	51	4	38	51	33	51	51
	**5**	1	8	18	0	3	18	9	23	25
**Endocrine disorders**	**1**	7	2	37	28	39	1	36	1	0
	**2**	25	4	27	24	31	9	21	1	1
	**3**	24	35	23	21	22	27	7	8	6
	**4**	18	42	3	2	3	39	4	29	25
	**5**	1	13	3	0	1	17	7	56	61
**Malignancy**	**1**	17	9	8	38	24	20	0	1	1
	**2**	35	25	20	24	44	21	6	0	0
	**3**	20	43	24	13	21	30	6	13	9
	**4**	3	17	30	0	5	15	26	39	30
	**5**	0	2	11	0	2	7	37	43	53
**Average total score**		**15.76**	**21.02**	**22.12**	**12.27**	**13.94**	**15.00**	**25.51**	**26.44**	**26.86**

Abbreviations:

UG–Undergraduate; JDO–Junior dental officer; SDO–Senior dental officer

Statistical analysis indicated that the UG had statistically significant lower confidence in the management of medically compromised patients (t-tests, UG vs JDO: p<0.00001, UG vs SDO: p<0.00001) and lower confidence in the management of an acute medical emergency in a dental setting (t-tests, UG vs JDO: p = 0.005, UG vs SDO: p = 0.0001)

When JDO and SDO were compared, no statistical differences were observed between their confidence and competence in the acute management of medical emergencies in a dental setting (t-tests, p = 0.51 and p = 0.10, respectively). However, the SDO were more confident in the management of medically compromised patients (t-test, p = 0.02).

### Section 3: Competence of the respondents

Both the JDO and SDO outperformed the UG group (MCQs competence scores of 25.51, 26.44. 26.86, respectively). This was consistent across all six domains. The mean MCQ score for the UG, JDO and SDO were 4.25, 4.41 and 4.48, respectively. If the passing score was considered to be 50%, only one JDO (1/264) failed. Only four UG, two JDO and one SDO did not manage to achieve a high pass grade of ≥70%. Statistical analysis indicated that the UG had significantly lower competence as compared to the JDOs and SDO (t-tests, UG vs JDO: p = 0.007, UG vs SDO: p<0.00009).

### Section 4: Perception of training

More than half of the respondents (61.7%) reported that a theory-only training provided by the dental school did not adequately prepare them for the management of medically compromised patients. These sentiments were shared across all three groups (Chi-square test, p<0.025). A statistically significant greater proportion of SDO (80.6%) felt that their dental officer postings prepared them to manage these patients compared to JDO (54.2%) (Chi-square, p<0.0001). Respondents who felt that their dental officer posting adequately prepared them to manage medical compromised patients had greater confidence in managing these patients and acute management of medical emergencies in dental setting. They also scored better for the MCQs (t-tests, p = 0.009, p<0.00001, p<0.002, respectively). The responses were summarised and can be found in Table 5.

**Table 5:** Perspective on dental school and post-graduation workplaces.

		UG	JDO	SDO
**Did theory-only training in dental school adequately prepare you to manage medically compromised patients?**	**Yes**	25	47	29
	**No**	50	49	64
**Did the experiences from your post-graduation clinical practice prepare you to manage medically compromised patients?**	**Yes**	N/A	52	75
	**No**	N/A	44	18

Abbreviations:

UG–Undergraduate; JDO–Junior Dental officer; SDO–Senior Dental officer

## Discussion

This study aimed to determine whether experiential learning is an important component of preparing dentists to manage medically compromised patients and acute medical emergencies in a dental setting. The results of this study suggested that theoretical training must be supplemented by experiential learning.

In general, the graduated dentists (JDO and SDO) were found to have greater confidence and competence when compared to the UG. With respect to the teaching of medically complex patients and medical emergencies, all three groups would have undergone similar theoretical training during dental school as there were no changes to the curriculum during their education period. The differences were that the JDO and SDO had an additional 1–2 and 3–4 years of clinical practice upon graduation, respectively. During this time period, both the JDO and SDO were more frequently exposed to medically compromised patients as compared to that of UG. This allowed the JDO and SDO to apply the theoretical knowledge into an “actual” clinical setting [1]. With each patient, the graduate can also reflect upon the experience, contextualize theoretical knowledge into the actual clinical scenarios and further improve their clinical practice [27, 28]. In a study by Watters et al, students were surveyed before and after a clinical rotation in the management of medically complex patients. The authors reported statistically significant increase in the comfort and interest in management of these patients after the clinical rotation. This highlights the importance of supplementing clinical training to theory lessons [29].

The results from the study also suggested that SDO were more confident than the JDO in the management of medically complex patients. This is not only attributed to the difference in the years of clinical practice but also, the type of patients seen in their practices and the access to consultation with various on-site or in-house dental specialists. Among the dental officers, 17.7% and 84.9% of the JDO and SDO, respectively, had experience working in a specialist outpatient clinic (SOC) or a multi-specialist service (MSS) (Table 1). The SOC and MSS are typically hospital-based practices or national centers which receive a greater clinical caseload and complexity of medically compromised patients. This is concurred by a significant amount of SDOs reporting that their dental officer postings have prepared them for the management of medically complex patients.

In contrast, there were no differences between the JDO and SDO in their confidence in the management of acute medical emergencies. This was despite the differences in experience as mentioned previously. This may be attributed to a generally low incidence of acute medical emergencies in a dental setting and thus less familiarity towards the necessary management. This is consistent with many prior studies examining the confidence of dental students or new graduates in the management of acute medical emergencies [15, 30–32]. For instance, Atherton et al reported that 97% of the dental staff from an UK hospital expressed the need for further training in the management of acute medical emergencies in a dental setting and this may be due to the low incidence of most acute medical emergencies at 1.8 events per years [22].

About 62% of the respondents agreed that theoretical training alone was not adequate preparation for the management of medically compromised patients and acute medical emergencies. This concern is not unique to our respondents [6, 15, 16, 21–23, 33, 34]. With limited time in the dental curriculum, some schools may prefer to focus more on other aspects of dentistry and place less emphasis on this module. However, the results of this study have already elicited the implications of forgoing clinical training in the management of these patients. Furthermore, with an increased burden of diseases that come from non-communicable, or chronic disease such as cardiovascular disorders and stroke, dental professionals can anticipate a greater proportion of medically compromised patients in their practice [13]. In addition, the public expects dentists to understand their medical conditions and handle acute medical emergencies in the dental setting [35]. Thus, it is imperative that a dental professional has sufficient knowledge to manage these patients and be adequately prepared to handle medical emergencies.

Most of the respondents of this study are currently working in the public service. Dental graduates are bonded to the public service for 4–5 years following the completion of a subsidized dental school education in Singapore. To our best knowledge, this scenario is unique to Singapore. In most nations, there is no mandatory public service of such extended duration for dental graduates. Other exceptions include a short 1-year internship at public dental services for fresh graduates in China, Japan, Belgium, Croatia and Slovenia [5–7, 36–38]. Thus, most dentists around the world have the liberty to enter private practice immediately after graduation, or at least much earlier in their career compared to Singapore-trained dentists. During the 4-year bond, Singapore dental graduates work under the supervision of senior colleagues who provide guidance and are available for case consultations. This serves as an opportunity for experiential learning in the management of medically compromised patients. Nonetheless, the presence of this extended bond and “training” should not discourage educators from improving the undergraduate education.

This study is not without limitations. Firstly, the ideal sample size for the UG group should be 84. However, we have failed to recruit sufficient dental students in the UG group. Although the number of respondents still made up more than 80% of the expected population, the results related to the UG group should still be interpreted with caution. Secondly, the male to female respondent ratio was not equal. The female gender made up 60% of the respondents. Although it was more likely for females to participate in an online survey, the skew in gender ratio reflects the actual gender ratio in the dental school in Singapore [39]. In the recent years, there are generally more female than male students. Thirdly, this is a cross-sectional study that reflected on the ability of students and graduates at a time point. The confidence and competence of the students and graduates may change over time. Lastly, the competence of the respondents was based solely on their MCQ score. While MCQs can assess their ability to apply their knowledge, it may not always translate to their actual performance in a clinical setting [25, 26].

Despite the limitations, the unique mandatory bond period of Singapore dental graduates allowed for cohorts of students to be compared, before and after clinical experiences in managing medically complex patients or acute medical emergencies. All 3 groups had similar theoretical training and thus the UG group served as a good control for the study. Most importantly, the findings of this study serve as a reminder for dental educators and administrators to assess on the adequacy of training of dental students in this aspect. With only theoretical education and no experiential training, young dental graduates may feel inadequate and unconfident to see medically compromised patients in their practice [14, 15, 40]. This can result in a barrier to oral healthcare for these patients [32, 41]. Therefore, there is a need to relook at the importance of dedicated clinical training, or specialist consultation clinics for the undergraduates to improve their exposure in this aspect [42]. This perspective is also shared by the United States Commission on Dental Accreditation [43].

## Conclusion

Experiential learning from “real-life” clinical experiences is an essential component in developing graduates’ confidence and competence in the management of medically compromised patients and acute medical emergencies. Most of the respondents indicated that a theory-only education provided by their dental school was insufficient in preparing them for the management of these patients. Instead, clinical exposure via actual work experience may have played a greater role in developing their confidence and competence in this aspect. Therefore, the authors recommend incorporating specialized clinical sessions in which dental students are allowed to treat and manage medically compromised patients. Future studies may develop these dental undergraduate modules and determine the effectiveness of experiential learning in increasing the confidence and competency of dentists.

## Supporting information

S1 File Minimal underlying data. (PDF)
