# Supplementary material for: Cross sectional study on the competence and confidence of dental students and graduates in the management of medically compromised patients and acute medical emergencies
Source: PLoS One. 2023 Feb 15;18(2):e0281801. doi: 10.1371/journal.pone.0281801 (PMC9931094; doi:10.1371/journal.pone.0281801)
Supplement: S1 File — (PDF) [file pone.0281801.s001.pdf]

CONFIDE

GENDER	YEAR	Worked in SOC/MSS 1 = Yes, 0=No	Frequency of treating Medically Complex Patient 1= Never, 2= Infrequently, 3= Frequently, 4 = Everyworking day	Does theory only in dental school prepare student sufficient 1 = Yes, 2 = No	Does work experience prepare you? 1= Yes, 2 = No	Cardio-vascular	Respiratory
Male	2017.00	1.00	3.00	1.00	1.00	5.00	5.00
Male	2017.00	1.00	3.00	1.00	1.00	5.00	5.00
Male	2018.00	1.00	3.00	0.00	1.00	5.00	5.00
Male	2019.00	1.00	4.00	1.00	1.00	5.00	5.00
Female	2017.00	1.00	3.00	0.00	1.00	5.00	5.00
Female	2018.00	1.00	4.00	0.00	1.00	5.00	5.00
Female	2018.00	1.00	4.00	0.00	1.00	5.00	5.00
Male	2018.00	1.00	3.00	0.00	0.00	5.00	5.00
Female	2017.00	1.00	2.00	0.00	1.00	5.00	4.00
Female	2018.00	1.00	4.00	1.00	1.00	5.00	5.00
Female	2017.00	1.00	2.00	1.00	1.00	5.00	5.00
Female	2019.00	0.00	4.00	0.00	1.00	5.00	5.00
Female	2018.00	1.00	4.00	0.00	1.00	5.00	5.00
Female	2018.00	1.00	4.00	0.00	1.00	5.00	5.00
Female	2018.00	1.00	4.00	0.00	1.00	5.00	5.00
Female	2019.00	0.00	4.00	0.00	0.00	4.00	5.00
Male	2019.00	0.00	3.00	1.00	1.00	3.00	3.00
Male	2018.00	1.00	3.00	0.00	1.00	5.00	4.00
Female	2018.00	1.00	3.00	0.00	1.00	4.00	4.00
Female	2018.00	1.00	4.00	0.00	1.00	4.00	5.00
Female	2019.00	0.00	4.00	0.00	0.00	5.00	5.00
Female	2019.00	0.00	4.00	0.00	0.00	5.00	5.00
Female	2017.00	1.00	3.00	0.00	1.00	5.00	4.00
Female	2017.00	1.00	3.00	0.00	1.00	4.00	4.00
Male	2019.00	0.00	3.00	1.00	0.00	5.00	5.00
Male	2019.00	0.00	3.00	1.00	0.00	5.00	5.00
Female	2018.00	1.00	3.00	0.00	1.00	5.00	5.00
Female	2019.00	0.00	3.00	1.00	1.00	4.00	5.00
Female	2019.00	0.00	3.00	0.00	1.00	4.00	5.00
Female	2019.00	0.00	3.00	0.00	1.00	4.00	5.00
Male	2020.00	0.00	3.00	1.00	1.00	4.00	4.00
Male	2018.00	1.00	4.00	0.00	1.00	4.00	4.00
Female	2018.00	1.00	4.00	0.00	1.00	5.00	4.00
Female	2019.00	0.00	3.00	0.00	1.00	4.00	4.00
Female	2017.00	1.00	4.00	0.00	1.00	5.00	4.00
Female	2017.00	0.00	2.00	0.00	1.00	5.00	4.00
Male	2019.00	0.00	2.00	1.00	0.00	4.00	4.00
Female	2017.00	1.00	2.00	0.00	1.00	4.00	5.00
Female	2017.00	1.00	2.00	0.00	1.00	4.00	5.00
Male	2017.00	1.00	2.00	1.00	1.00	4.00	4.00
Female	2019.00	0.00	4.00	1.00	1.00	4.00	4.00
Female	2017.00	1.00	3.00	0.00	1.00	4.00	4.00
Female	2017.00	1.00	3.00	0.00	1.00	4.00	5.00
Female	2019.00	0.00	3.00	0.00	1.00	4.00	4.00
Female	2019.00	0.00	3.00	1.00	1.00	4.00	4.00
Female	2020.00	1.00	2.00	1.00	1.00	4.00	4.00
Female	2020.00	1.00	2.00	1.00	1.00	4.00	4.00
Female	2019.00	0.00	3.00	1.00	0.00	4.00	4.00
Male	2020.00	0.00	4.00	1.00	1.00	4.00	4.00
Male	2020.00	1.00	4.00	0.00	1.00	4.00	4.00
Male	2019.00	0.00	2.00	1.00	1.00	4.00	4.00
Male	2018.00	1.00	4.00	0.00	1.00	5.00	4.00
Male	2019.00	0.00	2.00	1.00	0.00	4.00	4.00
Male	2019.00	0.00	2.00	1.00	0.00	4.00	4.00
Female	2017.00	1.00	4.00	1.00	1.00	4.00	4.00
Female	2017.00	1.00	4.00	1.00	1.00	4.00	4.00
Female	2017.00	1.00	4.00	1.00	1.00	4.00	4.00
Female	2020.00	1.00	3.00	0.00	1.00	4.00	4.00
Female	2018.00	1.00	4.00	0.00	1.00	4.00	4.00
Male	2017.00	1.00	3.00	0.00	1.00	5.00	3.00
Female	2017.00	1.00	3.00	0.00	1.00	5.00	4.00
Female	2017.00	1.00	3.00	0.00	1.00	5.00	4.00

Female	2020.00	1.00	4.00	1.00	1.00	4.00	4.00
Female	2020.00	1.00	4.00	1.00	1.00	4.00	4.00
Female	2020.00	1.00	4.00	1.00	1.00	4.00	4.00
Female	2019.00	0.00	3.00	0.00	1.00	5.00	4.00
Female	2017.00	1.00	2.00	0.00	1.00	4.00	4.00
Male	2017.00	1.00	3.00	0.00	1.00	4.00	3.00
Male	2017.00	1.00	3.00	0.00	1.00	4.00	3.00
Male	2017.00	1.00	3.00	0.00	1.00	4.00	4.00
Female	2018.00	1.00	3.00	1.00	1.00	4.00	4.00
Female	2019.00	0.00	2.00	0.00	0.00	4.00	4.00
Male	2019.00	0.00	2.00	0.00	0.00	4.00	4.00
Male	2019.00	0.00	3.00	1.00	0.00	4.00	4.00
Female	2019.00	0.00	3.00	1.00	0.00	4.00	4.00
Male	2019.00	0.00	3.00	1.00	1.00	4.00	4.00
Female	2019.00	0.00	3.00	0.00	0.00	4.00	4.00
Female	2019.00	0.00	3.00	0.00	1.00	4.00	4.00
Male	UG YEAR	0.00	2.00	0.00	0.00	4.00	4.00
Male	UG YEAR	0.00	2.00	0.00	0.00	4.00	4.00
Male	UG YEAR	0.00	2.00	0.00	0.00	4.00	4.00
Male	UG YEAR	0.00	2.00	0.00	0.00	4.00	4.00
Female	2017.00	1.00	3.00	0.00	1.00	4.00	4.00
Male	UG YEAR	0.00	1.00	0.00	0.00	4.00	4.00
Male	2018.00	0.00	3.00	0.00	0.00	4.00	4.00
Male	2018.00	0.00	3.00	0.00	0.00	4.00	4.00
Female	2017.00	1.00	3.00	0.00	1.00	4.00	3.00
Female	UG YEAR	0.00	2.00	0.00	0.00	4.00	4.00
Male	2018.00	1.00	2.00	0.00	0.00	5.00	4.00
Female	2017.00	1.00	2.00	0.00	0.00	5.00	4.00
Male	2017.00	1.00	3.00	1.00	1.00	5.00	4.00
Female	2019.00	0.00	3.00	1.00	0.00	4.00	4.00
Female	2020.00	1.00	4.00	1.00	1.00	4.00	4.00
Female	2020.00	1.00	4.00	1.00	1.00	4.00	4.00
Female	2019.00	0.00	3.00	0.00	0.00	4.00	4.00
Female	UG YEAR	0.00	2.00	1.00		4.00	4.00
Female	2020.00	1.00	3.00	1.00	1.00	4.00	4.00
Female	2020.00	1.00	3.00	1.00	1.00	4.00	4.00
Female	2018.00	1.00	2.00	0.00	0.00	4.00	4.00
Male	2019.00	0.00	2.00	1.00	0.00	4.00	4.00
Female	2018.00	1.00	3.00	0.00	1.00	4.00	3.00
Female	2019.00	0.00	4.00	0.00	1.00	4.00	4.00
Female	2018.00	1.00	3.00	0.00	0.00	4.00	4.00
Female	2018.00	1.00	3.00	0.00	0.00	4.00	4.00
Female	2020.00	1.00	3.00	0.00	0.00	3.00	4.00
Female	2020.00	1.00	3.00	0.00	0.00	3.00	4.00
Female	2018.00	0.00	3.00	0.00	0.00	4.00	4.00
Female	2020.00	1.00	4.00	1.00	1.00	4.00	4.00
Female	UG YEAR	0.00	2.00	0.00		4.00	3.00
Female	2018.00	1.00	3.00	0.00	1.00	3.00	4.00
Male	UG YEAR	0.00	2.00	1.00	0.00	3.00	3.00
Male	UG YEAR	0.00	2.00	1.00	0.00	3.00	3.00
Male	2020.00	0.00	1.00	1.00	0.00	4.00	4.00
Male	2020.00	0.00	1.00	1.00	0.00	4.00	4.00
Male	2020.00	0.00	1.00	1.00	0.00	4.00	4.00
Female	2018.00	1.00	4.00	0.00	1.00	5.00	4.00
Male	2020.00	1.00	2.00	0.00	0.00	4.00	3.00
Female	UG YEAR	0.00	3.00	1.00	1.00	4.00	4.00
Female	UG YEAR	0.00	3.00	1.00	1.00	4.00	4.00
Female	UG YEAR	0.00	3.00	1.00	1.00	4.00	4.00
Female	UG YEAR	0.00	3.00	1.00	1.00	4.00	4.00
Female	UG YEAR	0.00	3.00	1.00	1.00	4.00	4.00
Female	UG YEAR	0.00	3.00	1.00	1.00	4.00	4.00
Female	UG YEAR	0.00	3.00	1.00	1.00	4.00	4.00
Female	UG YEAR	0.00	3.00	1.00	1.00	4.00	4.00
Female	UG YEAR	0.00	2.00	0.00	0.00	3.00	4.00
Male	UG YEAR	0.00	2.00	0.00	0.00	3.00	4.00
Male	UG YEAR	0.00	2.00	0.00	0.00	3.00	4.00
Male	UG YEAR	0.00	2.00	0.00	0.00	3.00	4.00
Male	UG YEAR	0.00	2.00	0.00	0.00	3.00	4.00
Female	2019.00	0.00	3.00	0.00	1.00	4.00	3.00
Female	2018.00	0.00	2.00	0.00	1.00	5.00	5.00
Female	2019.00	0.00	4.00	0.00	1.00	5.00	3.00
Female	2017.00	1.00	2.00	1.00	1.00	4.00	4.00
Female	2018.00	1.00	3.00	1.00	1.00	3.00	4.00
Female	2018.00	1.00	3.00	1.00	1.00	3.00	4.00
Female	UG YEAR	0.00	2.00	1.00		4.00	5.00
Female	2018.00	0.00	2.00	1.00	1.00	4.00	4.00
Female	2020.00	1.00	2.00	0.00	1.00	4.00	4.00
Female	2020.00	1.00	2.00	0.00	1.00	4.00	4.00
Male	UG YEAR	0.00	2.00	1.00	0.00	3.00	3.00
Male	2019.00	0.00	4.00	1.00	1.00	4.00	4.00
Female	2018.00	1.00	3.00	0.00	1.00	5.00	3.00

Male	2017.00	1.00	3.00	0.00	1.00	4.00	2.00
Female	UG YEAR	0.00	2.00	0.00	0.00	3.00	4.00
Male	2019.00	0.00	3.00	0.00	1.00	4.00	4.00
Male	2017.00	1.00	4.00	0.00	1.00	4.00	4.00
Female	2018.00	1.00	3.00	0.00	0.00	4.00	4.00
Female	UG YEAR	0.00	2.00	0.00		3.00	4.00
Male	2017.00	1.00	3.00	0.00	1.00	4.00	4.00
Female	2018.00	1.00	4.00	0.00	1.00	4.00	2.00
Female	2018.00	1.00	3.00	0.00	1.00	4.00	3.00
Female	2017.00	0.00	2.00	1.00	0.00	4.00	4.00
Female	2019.00	0.00	2.00	1.00	0.00	5.00	3.00
Female	2017.00	1.00	2.00	1.00	1.00	4.00	4.00
Female	2017.00	1.00	4.00	0.00	1.00	5.00	4.00
Female	2018.00	1.00	3.00	0.00	1.00	4.00	3.00
Female	UG YEAR	0.00	2.00	0.00	0.00	4.00	2.00
Female	2017.00	1.00	3.00	1.00	1.00	4.00	4.00
Female	2020	1.00	3.00	0.00	1.00	3.00	3.00
Female	2018.00	1.00	3.00	1.00	1.00	4.00	3.00
Male	2019.00	0.00	2.00	1.00	1.00	4.00	3.00
Male	UG YEAR	0.00	2.00	1.00	0.00	3.00	4.00
Female	2019.00	0.00	3.00	0.00	0.00	4.00	3.00
Female	2019.00	0.00	3.00	0.00	0.00	3.00	4.00
Female	2018.00	1.00	3.00	0.00	1.00	4.00	3.00
Female	2019.00	0.00	2.00	1.00	0.00	4.00	3.00
Female	2019.00	0.00	2.00	1.00	0.00	4.00	4.00
Male	2020.00	0.00	2.00	0.00	1.00	4.00	5.00
Male	2020.00	0.00	2.00	0.00	1.00	4.00	5.00
Male	2020.00	0.00	2.00	0.00	1.00	4.00	5.00
Male	2020.00	0.00	2.00	0.00	1.00	4.00	5.00
Female	2017.00	1.00	3.00	0.00	1.00	4.00	3.00
Female	2020.00	0.00	2.00	0.00	1.00	4.00	3.00
Female	2018.00	1.00	2.00	0.00	1.00	4.00	4.00
Female	2020.00	1.00	2.00	1.00	1.00	4.00	4.00
Female	2020.00	1.00	2.00	1.00	1.00	4.00	4.00
Female	2020.00	1.00	2.00	1.00	1.00	4.00	4.00
Male	2020.00	1.00	3.00	0.00	0.00	4.00	3.00
Male	2017.00	1.00	2.00	1.00	1.00	4.00	3.00
Male	UG YEAR	0.00	2.00	1.00	0.00	3.00	3.00
Male	UG YEAR	0.00	2.00	1.00	0.00	3.00	3.00
Male	2017.00	1.00	3.00	1.00	0.00	3.00	3.00
Male	2020	0.00	2.00	0.00	0.00	3.00	3.00
Female	2017.00	1.00	2.00	1.00	1.00	3.00	4.00
Female	2019.00	0.00	2.00	0.00	0.00	3.00	3.00
Male	UG YEAR	0.00	2.00	0.00	0.00	4.00	3.00
Male	2020	0.00	3.00	1.00	0.00	5.00	3.00
Female	2020.00	0.00	2.00	1.00	0.00	3.00	4.00
Male	2017.00	1.00	2.00	0.00	0.00	3.00	3.00
Female	2019.00	0.00	3.00	1.00	0.00	3.00	3.00
Female	2019.00	0.00	4.00	0.00	0.00	3.00	3.00
Female	2019.00	0.00	2.00	1.00	0.00	3.00	3.00
Male	UG YEAR	0.00	2.00	1.00	0.00	4.00	3.00
Male	2019.00	0.00	4.00	0.00	0.00	4.00	3.00
Male	2019.00	0.00	4.00	0.00	0.00	4.00	3.00
Female	2019.00	0.00	2.00	1.00	1.00	4.00	4.00
Male	2017.00	0.00	2.00	1.00	1.00	4.00	3.00
Female	2018.00	1.00	3.00	1.00	1.00	3.00	3.00
Male	2017.00	0.00	2.00	1.00	1.00	3.00	2.00
Male	2017.00	0.00	2.00	1.00	1.00	3.00	2.00
Male	UG YEAR	0.00	2.00	1.00		3.00	3.00
Female	UG YEAR	0.00	1.00	1.00	0.00	3.00	4.00
Male	2019.00	0.00	2.00	0.00	0.00	4.00	4.00
Male	UG YEAR	0.00	2.00	0.00	0.00	2.00	3.00
Male	2019.00	0.00	4.00	1.00	1.00	3.00	3.00
Female	2017.00	1.00	3.00	0.00	1.00	3.00	3.00
Male	2018.00	0.00	2.00	1.00	0.00	3.00	4.00
Male	2019.00	0.00	1.00	0.00	0.00	3.00	4.00
Male	UG YEAR	0.00	2.00	1.00	0.00	3.00	3.00
Male	2017.00	0.00	2.00	1.00	0.00	3.00	3.00
Male	UG YEAR	0.00	2.00	0.00	0.00	3.00	3.00
Male	2017.00	0.00	2.00	1.00	0.00	3.00	3.00
Male	2017	0.00	2.00	1.00	0.00	5.00	3.00
Male	UG YEAR	0.00	2.00	0.00	0.00	3.00	3.00
Female	2017.00	1.00	3.00	0.00	1.00	4.00	3.00
Male	2018.00	1.00	4.00	0.00	1.00	4.00	3.00
Male	2018.00	1.00	4.00	0.00	1.00	4.00	3.00
Female	2019.00	0.00	3.00	0.00	0.00	3.00	3.00
Female	UG YEAR	0.00	1.00	0.00	0.00	3.00	3.00
Male	2019.00	1.00	2.00	0.00	1.00	2.00	3.00
Male	2020.00	0.00	2.00	0.00	0.00	2.00	3.00
Female	UG YEAR	0.00	2.00	0.00	0.00	3.00	2.00
Female	2020.00	0.00	2.00	0.00	1.00	3.00	3.00

Female	UG YEAR	0.00	2.00	0.00	0.00	2.00	2.00
Female	UG YEAR	0.00	2.00	0.00	0.00	3.00	3.00
Female	UG YEAR	0.00	2.00	0.00	0.00	3.00	3.00
Male	2019.00	0.00	2.00	0.00	0.00	4.00	3.00
Male	2019.00	0.00	2.00	0.00	0.00	4.00	3.00
Female	2020.00	1.00	2.00	0.00	1.00	3.00	2.00
Male	2020.00	1.00	3.00	1.00	1.00	2.00	2.00
Male	UG YEAR	0.00	2.00	0.00	0.00	3.00	3.00
Male	2020.00	1.00	2.00	0.00	1.00	3.00	2.00
Female	UG YEAR	0.00	1.00	1.00		3.00	2.00
Female	UG YEAR	0.00	2.00	0.00		3.00	2.00
Female	UG YEAR	0.00	2.00	0.00	0.00	3.00	2.00
Female	UG YEAR	0.00	2.00	0.00	0.00	3.00	2.00
Female	UG YEAR	0.00	2.00	0.00	0.00	3.00	2.00
Female	UG YEAR	0.00	2.00	0.00		3.00	2.00
Male	UG YEAR	0.00	2.00	0.00	0.00	2.00	2.00
Male	UG YEAR	0.00	2.00	0.00	0.00	2.00	2.00
Male	UG YEAR	0.00	2.00	0.00	0.00	2.00	2.00
Female	UG YEAR	0.00	2.00	0.00	0.00	3.00	2.00
Female	UG YEAR	0.00	2.00	0.00	0.00	3.00	2.00
Female	2017.00	1.00	2.00	0.00	0.00	3.00	2.00
Female	UG YEAR	0.00	2.00	0.00	0.00	2.00	2.00
Female	UG YEAR	0.00	2.00	0.00	0.00	2.00	2.00
Female	UG YEAR	0.00	2.00	0.00	0.00	2.00	2.00
Female	UG YEAR	0.00	2.00	0.00	0.00	2.00	2.00
Female	UG YEAR	0.00	3.00	1.00	0.00	2.00	2.00
Female	UG YEAR	0.00	3.00	1.00	0.00	2.00	2.00
Male	UG YEAR	0.00	1.00	0.00	0.00	3.00	2.00
Male	UG YEAR	0.00	2.00	0.00	1.00	1.00	3.00
Male	UG YEAR	0.00	2.00	0.00	1.00	1.00	3.00
Female	UG YEAR	0.00	1.00	1.00	1.00	2.00	2.00
Female	UG YEAR	0.00	1.00	1.00	1.00	2.00	2.00
Male	UG YEAR	0.00	2.00	0.00	1.00	2.00	2.00
Female	UG YEAR	0.00	2.00	0.00	0.00	1.00	1.00
Female	UG YEAR	0.00	2.00	0.00	0.00	1.00	1.00
Female	UG YEAR	0.00	2.00	0.00	0.00	1.00	1.00
Female	UG YEAR	0.00	2.00	0.00	0.00	1.00	1.00
Female	UG YEAR	0.00	2.00	0.00	0.00	1.00	1.00
Female	UG YEAR	0.00	1.00	0.00	0.00	1.00	1.00
Female	UG YEAR	0.00	1.00	0.00	0.00	1.00	1.00

NCE IN MANAGING PATIENTS THE FOLLOWING CONDITIONS

Neurological	Bleeding Disorder	Endocrine	Cancer	Summation	1 = if > or = 15, 0 < 15	Cardio-vascular
5.00	5.00	5.00	5.00	30.00	1.00	5.00
5.00	5.00	5.00	5.00	30.00	1.00	5.00
5.00	5.00	5.00	5.00	30.00	1.00	3.00
5.00	5.00	5.00	5.00	30.00	1.00	3.00
5.00	5.00	5.00	5.00	30.00	1.00	2.00
5.00	4.00	5.00	5.00	29.00	1.00	3.00
5.00	4.00	5.00	5.00	29.00	1.00	3.00
4.00	5.00	4.00	5.00	28.00	1.00	3.00
5.00	4.00	5.00	4.00	27.00	1.00	2.00
4.00	5.00	4.00	4.00	27.00	1.00	3.00
4.00	5.00	4.00	4.00	27.00	1.00	2.00
4.00	5.00	5.00	3.00	27.00	1.00	2.00
4.00	4.00	5.00	4.00	27.00	1.00	1.00
4.00	4.00	5.00	4.00	27.00	1.00	1.00
4.00	4.00	5.00	4.00	27.00	1.00	1.00
4.00	5.00	5.00	4.00	27.00	1.00	1.00
5.00	5.00	5.00	5.00	26.00	1.00	3.00
4.00	5.00	4.00	4.00	26.00	1.00	4.00
4.00	5.00	5.00	4.00	26.00	1.00	4.00
4.00	5.00	4.00	4.00	26.00	1.00	2.00
5.00	4.00	5.00	2.00	26.00	1.00	4.00
5.00	4.00	5.00	2.00	26.00	1.00	4.00
5.00	4.00	4.00	4.00	26.00	1.00	1.00
4.00	5.00	5.00	4.00	26.00	1.00	1.00
3.00	4.00	5.00	4.00	26.00	1.00	1.00
3.00	4.00	5.00	4.00	26.00	1.00	1.00
5.00	5.00	1.00	5.00	26.00	1.00	1.00
5.00	5.00	3.00	3.00	25.00	1.00	3.00
4.00	4.00	4.00	4.00	25.00	1.00	3.00
4.00	4.00	5.00	3.00	25.00	1.00	3.00
4.00	5.00	5.00	3.00	25.00	1.00	3.00
4.00	5.00	4.00	4.00	25.00	1.00	2.00
4.00	4.00	4.00	4.00	25.00	1.00	2.00
4.00	4.00	5.00	4.00	25.00	1.00	2.00
4.00	4.00	4.00	4.00	25.00	1.00	1.00
3.00	4.00	5.00	4.00	25.00	1.00	2.00
4.00	4.00	4.00	4.00	24.00	1.00	4.00
2.00	5.00	5.00	3.00	24.00	1.00	3.00
2.00	5.00	5.00	3.00	24.00	1.00	3.00
4.00	4.00	4.00	4.00	24.00	1.00	3.00
4.00	5.00	4.00	3.00	24.00	1.00	3.00
4.00	4.00	4.00	4.00	24.00	1.00	2.00
4.00	4.00	4.00	3.00	24.00	1.00	2.00
4.00	4.00	4.00	4.00	24.00	1.00	1.00
4.00	4.00	4.00	4.00	24.00	1.00	2.00
4.00	4.00	4.00	4.00	24.00	1.00	2.00
4.00	4.00	4.00	4.00	24.00	1.00	2.00
4.00	4.00	4.00	4.00	24.00	1.00	2.00
4.00	5.00	4.00	3.00	24.00	1.00	1.00
4.00	4.00	4.00	4.00	24.00	1.00	1.00
4.00	4.00	4.00	3.00	23.00	1.00	4.00
3.00	4.00	4.00	3.00	23.00	1.00	3.00
4.00	4.00	4.00	3.00	23.00	1.00	3.00
4.00	4.00	4.00	3.00	23.00	1.00	3.00
3.00	4.00	4.00	4.00	23.00	1.00	3.00
3.00	4.00	4.00	4.00	23.00	1.00	3.00
3.00	4.00	4.00	4.00	23.00	1.00	3.00
4.00	4.00	4.00	3.00	23.00	1.00	3.00
4.00	4.00	4.00	3.00	23.00	1.00	3.00
3.00	4.00	3.00	5.00	23.00	1.00	2.00
3.00	4.00	3.00	4.00	23.00	1.00	3.00
3.00	4.00	3.00	4.00	23.00	1.00	3.00

4.00	4.00	3.00	4.00	23.00	1.00	2.00
4.00	4.00	3.00	4.00	23.00	1.00	2.00
4.00	4.00	3.00	4.00	23.00	1.00	2.00
4.00	4.00	4.00	2.00	23.00	1.00	2.00
4.00	4.00	4.00	3.00	23.00	1.00	2.00
3.00	4.00	4.00	5.00	23.00	1.00	1.00
3.00	4.00	4.00	5.00	23.00	1.00	1.00
4.00	4.00	3.00	4.00	23.00	1.00	1.00
4.00	4.00	4.00	3.00	23.00	1.00	2.00
4.00	4.00	4.00	3.00	23.00	1.00	1.00
4.00	4.00	4.00	3.00	23.00	1.00	1.00
4.00	4.00	4.00	3.00	23.00	1.00	2.00
4.00	4.00	4.00	3.00	23.00	1.00	1.00
4.00	4.00	4.00	3.00	23.00	1.00	1.00
4.00	4.00	4.00	3.00	23.00	1.00	2.00
4.00	4.00	4.00	3.00	23.00	1.00	1.00
4.00	4.00	4.00	3.00	23.00	1.00	2.00
4.00	4.00	4.00	3.00	23.00	1.00	2.00
4.00	4.00	4.00	3.00	23.00	1.00	2.00
4.00	4.00	4.00	3.00	23.00	1.00	2.00
3.00	4.00	4.00	4.00	23.00	1.00	3.00
4.00	4.00	4.00	3.00	23.00	1.00	2.00
2.00	5.00	4.00	4.00	23.00	1.00	1.00
2.00	5.00	4.00	4.00	23.00	1.00	1.00
4.00	4.00	4.00	3.00	22.00	1.00	3.00
4.00	3.00	3.00	4.00	22.00	1.00	3.00
2.00	4.00	3.00	4.00	22.00	1.00	2.00
4.00	4.00	4.00	1.00	22.00	1.00	2.00
3.00	5.00	4.00	1.00	22.00	1.00	1.00
4.00	4.00	4.00	2.00	22.00	1.00	1.00
3.00	4.00	4.00	3.00	22.00	1.00	1.00
3.00	4.00	4.00	3.00	22.00	1.00	1.00
3.00	4.00	4.00	3.00	22.00	1.00	3.00
4.00	3.00	5.00	2.00	22.00	1.00	2.00
3.00	4.00	4.00	3.00	22.00	1.00	1.00
3.00	4.00	4.00	3.00	22.00	1.00	1.00
4.00	4.00	4.00	2.00	22.00	1.00	2.00
3.00	4.00	4.00	3.00	22.00	1.00	2.00
4.00	4.00	4.00	3.00	22.00	1.00	1.00
3.00	4.00	5.00	2.00	22.00	1.00	1.00
3.00	3.00	4.00	4.00	22.00	1.00	2.00
3.00	3.00	4.00	4.00	22.00	1.00	2.00
4.00	3.00	4.00	4.00	22.00	1.00	1.00
4.00	3.00	4.00	4.00	22.00	1.00	1.00
4.00	4.00	4.00	2.00	22.00	1.00	1.00
3.00	4.00	5.00	2.00	22.00	1.00	1.00
4.00	3.00	4.00	3.00	21.00	1.00	4.00
3.00	4.00	4.00	3.00	21.00	1.00	3.00
3.00	4.00	4.00	4.00	21.00	1.00	2.00
3.00	4.00	4.00	4.00	21.00	1.00	2.00
3.00	4.00	3.00	3.00	21.00	1.00	4.00
3.00	4.00	3.00	3.00	21.00	1.00	4.00
3.00	4.00	3.00	3.00	21.00	1.00	4.00
4.00	4.00	2.00	2.00	21.00	1.00	3.00
4.00	4.00	4.00	2.00	21.00	1.00	2.00
4.00	4.00	3.00	2.00	21.00	1.00	4.00
4.00	4.00	3.00	2.00	21.00	1.00	4.00
4.00	4.00	3.00	2.00	21.00	1.00	4.00
4.00	4.00	3.00	2.00	21.00	1.00	4.00
4.00	4.00	3.00	2.00	21.00	1.00	4.00
4.00	4.00	3.00	2.00	21.00	1.00	4.00
4.00	4.00	3.00	2.00	21.00	1.00	4.00
4.00	4.00	3.00	2.00	21.00	1.00	4.00
3.00	4.00	4.00	3.00	21.00	1.00	2.00
3.00	4.00	4.00	3.00	21.00	1.00	2.00
3.00	4.00	4.00	3.00	21.00	1.00	2.00
3.00	4.00	4.00	3.00	21.00	1.00	2.00
3.00	4.00	4.00	3.00	21.00	1.00	2.00
3.00	4.00	4.00	3.00	21.00	1.00	2.00
3.00	3.00	3.00	2.00	21.00	1.00	1.00
4.00	4.00	3.00	2.00	21.00	1.00	1.00
3.00	4.00	3.00	3.00	21.00	1.00	1.00
4.00	4.00	4.00	2.00	21.00	1.00	2.00
4.00	4.00	4.00	2.00	21.00	1.00	2.00
1.00	5.00	3.00	3.00	21.00	1.00	2.00
3.00	3.00	4.00	3.00	21.00	1.00	1.00
3.00	3.00	4.00	3.00	21.00	1.00	1.00
3.00	3.00	4.00	3.00	21.00	1.00	1.00
4.00	4.00	3.00	3.00	20.00	1.00	4.00
3.00	4.00	4.00	1.00	20.00	1.00	4.00
2.00	4.00	3.00	3.00	20.00	1.00	3.00

3.00	4.00	3.00	4.00	20.00	1.00	2.00
3.00	3.00	4.00	3.00	20.00	1.00	2.00
3.00	3.00	3.00	3.00	20.00	1.00	2.00
2.00	4.00	3.00	3.00	20.00	1.00	2.00
3.00	3.00	3.00	3.00	20.00	1.00	2.00
3.00	3.00	4.00	3.00	20.00	1.00	2.00
3.00	4.00	2.00	3.00	20.00	1.00	2.00
4.00	4.00	3.00	3.00	20.00	1.00	3.00
3.00	4.00	4.00	2.00	20.00	1.00	2.00
3.00	4.00	3.00	2.00	20.00	1.00	2.00
3.00	3.00	3.00	3.00	20.00	1.00	1.00
4.00	2.00	5.00	1.00	20.00	1.00	1.00
2.00	3.00	3.00	3.00	20.00	1.00	1.00
3.00	4.00	3.00	3.00	20.00	1.00	2.00
3.00	4.00	4.00	3.00	20.00	1.00	1.00
4.00	3.00	4.00	1.00	20.00	1.00	1.00
3.00	4.00	3.00	3.00	19.00	1.00	3.00
3.00	3.00	3.00	3.00	19.00	1.00	4.00
3.00	3.00	3.00	3.00	19.00	1.00	4.00
4.00	3.00	3.00	2.00	19.00	1.00	4.00
3.00	3.00	3.00	3.00	19.00	1.00	3.00
3.00	3.00	3.00	3.00	19.00	1.00	1.00
3.00	3.00	3.00	3.00	19.00	1.00	2.00
3.00	3.00	3.00	3.00	19.00	1.00	1.00
2.00	3.00	4.00	2.00	19.00	1.00	2.00
3.00	3.00	3.00	1.00	19.00	1.00	2.00
3.00	3.00	3.00	1.00	19.00	1.00	2.00
3.00	3.00	3.00	1.00	19.00	1.00	2.00
3.00	3.00	3.00	1.00	19.00	1.00	2.00
3.00	4.00	3.00	2.00	19.00	1.00	2.00
3.00	3.00	4.00	2.00	19.00	1.00	1.00
3.00	4.00	3.00	1.00	19.00	1.00	1.00
3.00	3.00	3.00	2.00	19.00	1.00	1.00
3.00	3.00	3.00	2.00	19.00	1.00	1.00
3.00	3.00	3.00	2.00	19.00	1.00	1.00
2.00	4.00	4.00	2.00	19.00	1.00	1.00
2.00	3.00	3.00	3.00	18.00	1.00	3.00
3.00	3.00	3.00	3.00	18.00	1.00	3.00
3.00	3.00	3.00	3.00	18.00	1.00	3.00
3.00	3.00	3.00	3.00	18.00	1.00	3.00
3.00	3.00	3.00	3.00	18.00	1.00	3.00
3.00	3.00	3.00	2.00	18.00	1.00	2.00
3.00	3.00	3.00	3.00	18.00	1.00	2.00
2.00	3.00	3.00	3.00	18.00	1.00	3.00
2.00	3.00	3.00	2.00	18.00	1.00	3.00
3.00	3.00	3.00	2.00	18.00	1.00	2.00
3.00	4.00	3.00	2.00	18.00	1.00	2.00
3.00	3.00	3.00	3.00	18.00	1.00	2.00
3.00	3.00	3.00	3.00	18.00	1.00	2.00
3.00	3.00	3.00	3.00	18.00	1.00	2.00
3.00	3.00	3.00	2.00	18.00	1.00	2.00
2.00	3.00	3.00	3.00	18.00	1.00	2.00
2.00	3.00	3.00	3.00	18.00	1.00	2.00
3.00	3.00	2.00	2.00	18.00	1.00	1.00
3.00	2.00	5.00	1.00	18.00	1.00	1.00
3.00	3.00	3.00	3.00	18.00	1.00	1.00
4.00	4.00	2.00	2.00	17.00	1.00	2.00
4.00	4.00	2.00	2.00	17.00	1.00	2.00
3.00	3.00	3.00	2.00	17.00	1.00	3.00
3.00	3.00	2.00	2.00	17.00	1.00	2.00
1.00	3.00	4.00	1.00	17.00	1.00	2.00
3.00	3.00	4.00	2.00	17.00	1.00	2.00
3.00	3.00	3.00	2.00	17.00	1.00	1.00
3.00	3.00	3.00	2.00	17.00	1.00	1.00
2.00	3.00	4.00	1.00	17.00	1.00	1.00
2.00	4.00	3.00	1.00	17.00	1.00	1.00
2.00	3.00	3.00	2.00	16.00	1.00	3.00
2.00	3.00	3.00	2.00	16.00	1.00	2.00
2.00	3.00	3.00	2.00	16.00	1.00	2.00
2.00	3.00	3.00	2.00	16.00	1.00	2.00
2.00	2.00	2.00	2.00	16.00	1.00	2.00
2.00	3.00	2.00	3.00	16.00	1.00	2.00
2.00	3.00	2.00	2.00	16.00	1.00	1.00
2.00	3.00	2.00	2.00	16.00	1.00	1.00
2.00	3.00	2.00	2.00	16.00	1.00	1.00
2.00	2.00	4.00	2.00	16.00	1.00	1.00
3.00	3.00	3.00	1.00	16.00	1.00	1.00
3.00	2.00	3.00	2.00	15.00	1.00	2.00
2.00	3.00	3.00	2.00	15.00	1.00	2.00
3.00	3.00	3.00	1.00	15.00	1.00	2.00
3.00	3.00	1.00	2.00	15.00	1.00	2.00

2.00	3.00	4.00	2.00	15.00	1.00	1.00
2.00	2.00	3.00	2.00	15.00	1.00	2.00
2.00	2.00	3.00	2.00	15.00	1.00	2.00
2.00	3.00	2.00	1.00	15.00	1.00	1.00
2.00	3.00	2.00	1.00	15.00	1.00	1.00
2.00	3.00	2.00	2.00	14.00	0.00	2.00
2.00	2.00	4.00	2.00	14.00	0.00	1.00
2.00	2.00	2.00	2.00	14.00	0.00	1.00
1.00	4.00	1.00	2.00	13.00	0.00	1.00
2.00	2.00	2.00	2.00	13.00	0.00	2.00
1.00	2.00	3.00	2.00	13.00	0.00	1.00
2.00	2.00	2.00	2.00	13.00	0.00	2.00
2.00	2.00	2.00	2.00	13.00	0.00	2.00
2.00	2.00	2.00	2.00	13.00	0.00	2.00
2.00	2.00	2.00	2.00	13.00	0.00	1.00
2.00	2.00	2.00	2.00	12.00	0.00	2.00
2.00	2.00	2.00	2.00	12.00	0.00	2.00
1.00	3.00	2.00	2.00	12.00	0.00	2.00
1.00	2.00	2.00	2.00	12.00	0.00	1.00
1.00	2.00	2.00	2.00	12.00	0.00	1.00
2.00	2.00	2.00	1.00	12.00	0.00	1.00
1.00	2.00	2.00	2.00	11.00	0.00	2.00
1.00	2.00	2.00	2.00	11.00	0.00	2.00
1.00	2.00	2.00	2.00	11.00	0.00	2.00
1.00	2.00	2.00	2.00	11.00	0.00	2.00
2.00	2.00	2.00	1.00	11.00	0.00	1.00
2.00	2.00	2.00	1.00	11.00	0.00	1.00
2.00	1.00	2.00	1.00	11.00	0.00	1.00
1.00	2.00	1.00	1.00	9.00	0.00	1.00
1.00	2.00	1.00	1.00	9.00	0.00	1.00
1.00	2.00	1.00	1.00	9.00	0.00	1.00
1.00	2.00	1.00	1.00	9.00	0.00	1.00
1.00	1.00	1.00	1.00	8.00	0.00	1.00
1.00	1.00	2.00	1.00	7.00	0.00	1.00
1.00	1.00	2.00	1.00	7.00	0.00	1.00
1.00	1.00	2.00	1.00	7.00	0.00	1.00
1.00	1.00	2.00	1.00	7.00	0.00	1.00
1.00	1.00	2.00	1.00	7.00	0.00	1.00
1.00	1.00	1.00	1.00	6.00	0.00	1.00
1.00	1.00	1.00	1.00	6.00	0.00	1.00

CONFIDENCE IN MANAGING THE FOLLOWING EMERGENCIES

Respiratory	Neurological	Bleeding Disorder	Endocrine	Cancer	Summation	1 = if > or = 15, 0 < 15
5.00	5.00	5.00	5.00	5.00	30.00	1.00
5.00	5.00	5.00	5.00	5.00	30.00	1.00
5.00	5.00	5.00	5.00	5.00	28.00	1.00
3.00	4.00	5.00	1.00	5.00	21.00	1.00
2.00	2.00	5.00	1.00	5.00	17.00	1.00
3.00	3.00	4.00	3.00	3.00	19.00	1.00
3.00	3.00	4.00	3.00	3.00	19.00	1.00
4.00	3.00	5.00	4.00	4.00	23.00	1.00
4.00	4.00	4.00	4.00	4.00	22.00	1.00
4.00	3.00	4.00	2.00	3.00	19.00	1.00
3.00	2.00	4.00	3.00	3.00	17.00	1.00
4.00	2.00	4.00	3.00	2.00	17.00	1.00
1.00	1.00	3.00	1.00	3.00	10.00	0.00
1.00	1.00	3.00	1.00	3.00	10.00	0.00
1.00	1.00	3.00	1.00	3.00	10.00	0.00
1.00	1.00	3.00	1.00	1.00	8.00	0.00
3.00	5.00	5.00	5.00	5.00	26.00	1.00
4.00	4.00	5.00	4.00	4.00	25.00	1.00
3.00	3.00	4.00	3.00	4.00	21.00	1.00
4.00	3.00	4.00	3.00	3.00	19.00	1.00
3.00	3.00	4.00	1.00	2.00	17.00	1.00
3.00	3.00	4.00	1.00	2.00	17.00	1.00
2.00	2.00	4.00	2.00	4.00	15.00	1.00
2.00	1.00	4.00	3.00	4.00	15.00	1.00
3.00	1.00	4.00	1.00	2.00	12.00	0.00
3.00	1.00	4.00	1.00	2.00	12.00	0.00
1.00	1.00	2.00	1.00	1.00	7.00	0.00
5.00	5.00	5.00	1.00	2.00	21.00	1.00
3.00	3.00	4.00	3.00	3.00	19.00	1.00
4.00	3.00	4.00	4.00	1.00	19.00	1.00
2.00	3.00	4.00	3.00	2.00	17.00	1.00
3.00	2.00	4.00	3.00	3.00	17.00	1.00
2.00	2.00	4.00	1.00	4.00	15.00	1.00
2.00	2.00	4.00	1.00	3.00	14.00	0.00
2.00	1.00	4.00	2.00	3.00	13.00	0.00
3.00	2.00	4.00	1.00	1.00	13.00	0.00
4.00	4.00	4.00	4.00	4.00	24.00	1.00
3.00	3.00	5.00	2.00	3.00	19.00	1.00
3.00	3.00	5.00	2.00	3.00	19.00	1.00
3.00	3.00	4.00	3.00	3.00	19.00	1.00
3.00	3.00	4.00	3.00	3.00	19.00	1.00
2.00	4.00	4.00	3.00	3.00	18.00	1.00
3.00	2.00	4.00	3.00	3.00	17.00	1.00
3.00	2.00	4.00	1.00	4.00	15.00	1.00
2.00	3.00	3.00	3.00	2.00	15.00	1.00
2.00	2.00	3.00	2.00	3.00	14.00	0.00
2.00	2.00	3.00	2.00	3.00	14.00	0.00
2.00	2.00	3.00	2.00	1.00	12.00	0.00
1.00	1.00	4.00	1.00	3.00	11.00	0.00
2.00	2.00	2.00	2.00	1.00	10.00	0.00
4.00	4.00	4.00	2.00	3.00	21.00	1.00
3.00	3.00	5.00	3.00	3.00	20.00	1.00
4.00	4.00	4.00	3.00	2.00	20.00	1.00
4.00	4.00	4.00	3.00	2.00	20.00	1.00
3.00	2.00	4.00	3.00	4.00	19.00	1.00
3.00	2.00	4.00	3.00	4.00	19.00	1.00
3.00	2.00	4.00	3.00	4.00	19.00	1.00
3.00	3.00	4.00	3.00	3.00	19.00	1.00
3.00	3.00	4.00	2.00	3.00	18.00	1.00
2.00	2.00	4.00	3.00	5.00	18.00	1.00
2.00	3.00	4.00	2.00	4.00	18.00	1.00
2.00	3.00	4.00	2.00	4.00	18.00	1.00

2.00	3.00	4.00	2.00	4.00	17.00	1.00
2.00	3.00	4.00	2.00	4.00	17.00	1.00
2.00	3.00	4.00	2.00	4.00	17.00	1.00
3.00	3.00	4.00	2.00	3.00	17.00	1.00
2.00	3.00	4.00	3.00	2.00	16.00	1.00
1.00	2.00	5.00	2.00	5.00	16.00	1.00
1.00	2.00	5.00	2.00	5.00	16.00	1.00
2.00	3.00	4.00	1.00	4.00	15.00	1.00
3.00	2.00	4.00	2.00	2.00	15.00	1.00
2.00	3.00	3.00	2.00	3.00	14.00	0.00
2.00	3.00	3.00	2.00	3.00	14.00	0.00
2.00	2.00	4.00	2.00	2.00	14.00	0.00
3.00	2.00	3.00	3.00	1.00	13.00	0.00
3.00	3.00	3.00	2.00	1.00	13.00	0.00
2.00	2.00	3.00	2.00	2.00	13.00	0.00
2.00	2.00	2.00	3.00	2.00	12.00	0.00
2.00	2.00	3.00	2.00	1.00	12.00	0.00
2.00	2.00	3.00	2.00	1.00	12.00	0.00
2.00	2.00	3.00	2.00	1.00	12.00	0.00
2.00	2.00	3.00	2.00	1.00	12.00	0.00
2.00	1.00	2.00	2.00	1.00	11.00	0.00
3.00	1.00	1.00	3.00	1.00	11.00	0.00
1.00	1.00	3.00	1.00	1.00	8.00	0.00
1.00	1.00	3.00	1.00	1.00	8.00	0.00
4.00	2.00	4.00	2.00	3.00	18.00	1.00
3.00	3.00	3.00	3.00	3.00	18.00	1.00
3.00	2.00	3.00	1.00	3.00	14.00	0.00
2.00	2.00	4.00	2.00	2.00	14.00	0.00
3.00	3.00	5.00	1.00	1.00	14.00	0.00
3.00	1.00	3.00	3.00	3.00	14.00	0.00
2.00	2.00	4.00	2.00	2.00	13.00	0.00
2.00	2.00	4.00	2.00	2.00	13.00	0.00
3.00	2.00	2.00	1.00	2.00	13.00	0.00
2.00	2.00	3.00	2.00	2.00	13.00	0.00
2.00	1.00	4.00	1.00	3.00	12.00	0.00
2.00	1.00	4.00	1.00	3.00	12.00	0.00
2.00	2.00	3.00	2.00	1.00	12.00	0.00
3.00	2.00	3.00	1.00	1.00	12.00	0.00
1.00	1.00	4.00	3.00	2.00	12.00	0.00
2.00	1.00	4.00	1.00	2.00	11.00	0.00
1.00	1.00	3.00	1.00	3.00	11.00	0.00
1.00	1.00	3.00	1.00	3.00	11.00	0.00
1.00	2.00	3.00	1.00	2.00	10.00	0.00
1.00	2.00	3.00	1.00	2.00	10.00	0.00
2.00	1.00	3.00	1.00	1.00	9.00	0.00
1.00	1.00	3.00	1.00	1.00	8.00	0.00
3.00	4.00	3.00	3.00	3.00	20.00	1.00
3.00	4.00	4.00	3.00	3.00	20.00	1.00
4.00	4.00	3.00	3.00	3.00	19.00	1.00
4.00	4.00	3.00	3.00	3.00	19.00	1.00
3.00	3.00	3.00	3.00	2.00	18.00	1.00
3.00	3.00	3.00	3.00	2.00	18.00	1.00
3.00	3.00	3.00	3.00	2.00	18.00	1.00
4.00	3.00	4.00	1.00	3.00	18.00	1.00
3.00	4.00	4.00	4.00	1.00	18.00	1.00
3.00	3.00	3.00	3.00	2.00	18.00	1.00
3.00	3.00	3.00	3.00	2.00	18.00	1.00
3.00	3.00	3.00	3.00	2.00	18.00	1.00
3.00	3.00	3.00	3.00	2.00	18.00	1.00
3.00	3.00	3.00	3.00	2.00	18.00	1.00
3.00	3.00	3.00	3.00	2.00	18.00	1.00
3.00	3.00	3.00	3.00	2.00	18.00	1.00
3.00	3.00	3.00	3.00	2.00	18.00	1.00
3.00	2.00	3.00	4.00	3.00	17.00	1.00
3.00	2.00	3.00	3.00	3.00	16.00	1.00
3.00	2.00	3.00	3.00	3.00	16.00	1.00
3.00	2.00	3.00	3.00	3.00	16.00	1.00
3.00	2.00	3.00	3.00	3.00	16.00	1.00
3.00	2.00	4.00	2.00	2.00	15.00	1.00
2.00	1.00	4.00	2.00	4.00	14.00	0.00
2.00	3.00	4.00	2.00	2.00	14.00	0.00
3.00	2.00	3.00	2.00	2.00	13.00	0.00
2.00	2.00	4.00	1.00	2.00	13.00	0.00
2.00	2.00	4.00	1.00	2.00	13.00	0.00
2.00	2.00	3.00	1.00	2.00	12.00	0.00
2.00	1.00	2.00	1.00	2.00	9.00	0.00
1.00	1.00	2.00	1.00	1.00	7.00	0.00
1.00	1.00	2.00	1.00	1.00	7.00	0.00
4.00	4.00	4.00	4.00	3.00	23.00	1.00
4.00	4.00	4.00	3.00	1.00	20.00	1.00
2.00	2.00	5.00	3.00	3.00	18.00	1.00

3.00	2.00	4.00	1.00	4.00	16.00	1.00
3.00	2.00	4.00	2.00	3.00	16.00	1.00
2.00	3.00	3.00	3.00	3.00	16.00	1.00
2.00	2.00	4.00	2.00	3.00	15.00	1.00
3.00	1.00	4.00	2.00	3.00	15.00	1.00
3.00	2.00	3.00	2.00	2.00	14.00	0.00
2.00	2.00	4.00	1.00	2.00	13.00	0.00
3.00	1.00	3.00	1.00	2.00	13.00	0.00
2.00	2.00	4.00	1.00	2.00	13.00	0.00
2.00	2.00	2.00	3.00	2.00	13.00	0.00
1.00	2.00	3.00	2.00	3.00	12.00	0.00
2.00	1.00	5.00	1.00	1.00	11.00	0.00
1.00	1.00	4.00	2.00	1.00	10.00	0.00
1.00	2.00	3.00	1.00	1.00	10.00	0.00
1.00	3.00	2.00	1.00	1.00	9.00	0.00
1.00	1.00	3.00	1.00	1.00	8.00	0.00
3.00	3.00	4.00	3.00	3.00	19.00	1.00
3.00	3.00	3.00	3.00	3.00	19.00	1.00
3.00	3.00	3.00	3.00	3.00	19.00	1.00
4.00	4.00	3.00	2.00	2.00	19.00	1.00
3.00	2.00	4.00	1.00	3.00	16.00	1.00
2.00	3.00	3.00	3.00	3.00	15.00	1.00
2.00	2.00	3.00	2.00	3.00	14.00	0.00
2.00	2.00	3.00	2.00	2.00	12.00	0.00
2.00	2.00	3.00	1.00	2.00	12.00	0.00
3.00	2.00	2.00	1.00	2.00	12.00	0.00
3.00	2.00	2.00	1.00	2.00	12.00	0.00
3.00	2.00	2.00	1.00	2.00	12.00	0.00
3.00	2.00	2.00	1.00	2.00	12.00	0.00
1.00	1.00	4.00	1.00	2.00	11.00	0.00
2.00	1.00	4.00	1.00	1.00	10.00	0.00
2.00	1.00	3.00	2.00	1.00	10.00	0.00
2.00	1.00	3.00	1.00	1.00	9.00	0.00
2.00	1.00	3.00	1.00	1.00	9.00	0.00
2.00	1.00	3.00	1.00	1.00	9.00	0.00
1.00	1.00	3.00	1.00	2.00	9.00	0.00
3.00	3.00	3.00	3.00	3.00	18.00	1.00
3.00	3.00	3.00	3.00	3.00	18.00	1.00
3.00	3.00	3.00	3.00	3.00	18.00	1.00
3.00	3.00	3.00	3.00	3.00	18.00	1.00
3.00	3.00	3.00	2.00	3.00	17.00	1.00
3.00	3.00	4.00	2.00	2.00	16.00	1.00
3.00	3.00	3.00	3.00	2.00	16.00	1.00
3.00	2.00	3.00	3.00	2.00	16.00	1.00
3.00	2.00	3.00	3.00	2.00	16.00	1.00
2.00	2.00	4.00	2.00	2.00	14.00	0.00
3.00	2.00	3.00	2.00	2.00	14.00	0.00
3.00	2.00	3.00	2.00	2.00	14.00	0.00
3.00	2.00	3.00	1.00	2.00	13.00	0.00
3.00	1.00	3.00	2.00	2.00	13.00	0.00
2.00	2.00	2.00	2.00	2.00	12.00	0.00
2.00	1.00	4.00	2.00	1.00	12.00	0.00
2.00	1.00	4.00	2.00	1.00	12.00	0.00
1.00	2.00	3.00	2.00	2.00	11.00	0.00
1.00	1.00	5.00	1.00	1.00	10.00	0.00
1.00	1.00	3.00	1.00	1.00	8.00	0.00
5.00	3.00	5.00	1.00	2.00	18.00	1.00
5.00	3.00	5.00	1.00	2.00	18.00	1.00
2.00	3.00	4.00	2.00	2.00	16.00	1.00
2.00	2.00	4.00	2.00	2.00	14.00	0.00
2.00	2.00	3.00	3.00	1.00	13.00	0.00
3.00	2.00	2.00	1.00	2.00	12.00	0.00
2.00	2.00	3.00	1.00	2.00	11.00	0.00
2.00	2.00	3.00	1.00	1.00	10.00	0.00
1.00	1.00	2.00	1.00	1.00	7.00	0.00
1.00	1.00	2.00	1.00	1.00	7.00	0.00
3.00	3.00	3.00	3.00	2.00	17.00	1.00
2.00	2.00	4.00	2.00	2.00	14.00	0.00
3.00	2.00	3.00	2.00	2.00	14.00	0.00
2.00	2.00	4.00	2.00	2.00	14.00	0.00
2.00	2.00	2.00	2.00	2.00	12.00	0.00
3.00	3.00	2.00	1.00	1.00	12.00	0.00
2.00	2.00	3.00	1.00	2.00	11.00	0.00
1.00	1.00	2.00	1.00	1.00	7.00	0.00
1.00	1.00	2.00	1.00	1.00	7.00	0.00
1.00	1.00	1.00	1.00	1.00	6.00	0.00
1.00	1.00	1.00	1.00	1.00	6.00	0.00
2.00	2.00	3.00	2.00	2.00	13.00	0.00
2.00	2.00	3.00	2.00	2.00	13.00	0.00
2.00	2.00	3.00	2.00	2.00	13.00	0.00
2.00	2.00	2.00	1.00	2.00	11.00	0.00

2.00	1.00	3.00	2.00	1.00	10.00	0.00
2.00	1.00	2.00	1.00	1.00	9.00	0.00
2.00	1.00	2.00	1.00	1.00	9.00	0.00
1.00	1.00	3.00	1.00	1.00	8.00	0.00
1.00	1.00	3.00	1.00	1.00	8.00	0.00
2.00	2.00	2.00	2.00	2.00	12.00	0.00
3.00	2.00	2.00	2.00	1.00	11.00	0.00
1.00	1.00	1.00	1.00	1.00	6.00	0.00
3.00	3.00	4.00	1.00	2.00	14.00	0.00
2.00	2.00	2.00	2.00	2.00	12.00	0.00
2.00	2.00	3.00	1.00	1.00	10.00	0.00
2.00	1.00	3.00	1.00	1.00	10.00	0.00
2.00	1.00	3.00	1.00	1.00	10.00	0.00
2.00	1.00	3.00	1.00	1.00	10.00	0.00
1.00	1.00	2.00	1.00	1.00	7.00	0.00
2.00	2.00	2.00	2.00	2.00	12.00	0.00
2.00	2.00	1.00	1.00	1.00	9.00	0.00
2.00	1.00	2.00	1.00	1.00	9.00	0.00
1.00	1.00	2.00	2.00	2.00	9.00	0.00
1.00	1.00	2.00	2.00	2.00	9.00	0.00
1.00	1.00	3.00	1.00	1.00	8.00	0.00
2.00	1.00	2.00	2.00	1.00	10.00	0.00
2.00	1.00	2.00	2.00	1.00	10.00	0.00
2.00	1.00	2.00	2.00	1.00	10.00	0.00
2.00	1.00	2.00	2.00	1.00	10.00	0.00
2.00	1.00	3.00	2.00	1.00	10.00	0.00
2.00	1.00	3.00	2.00	1.00	10.00	0.00
1.00	1.00	1.00	1.00	1.00	6.00	0.00
2.00	1.00	2.00	1.00	1.00	8.00	0.00
2.00	1.00	2.00	1.00	1.00	8.00	0.00
1.00	1.00	2.00	1.00	1.00	7.00	0.00
1.00	1.00	2.00	1.00	1.00	7.00	0.00
1.00	1.00	2.00	1.00	1.00	7.00	0.00
1.00	1.00	1.00	1.00	1.00	6.00	0.00
1.00	1.00	1.00	1.00	1.00	6.00	0.00
1.00	1.00	1.00	1.00	1.00	6.00	0.00
1.00	1.00	1.00	1.00	1.00	6.00	0.00
1.00	1.00	1.00	1.00	1.00	6.00	0.00
1.00	1.00	1.00	1.00	1.00	6.00	0.00
1.00	1.00	1.00	1.00	1.00	6.00	0.00

Scores in each domain for competence

Cardio-vascular	Respiratory	Neurological	Bleeding Disorder	Endocrine	Cancer	Summation
5.00	5.00	5.00	5.00	5.00	5.00	30.00
5.00	5.00	5.00	5.00	5.00	5.00	30.00
4.00	3.00	5.00	3.00	5.00	4.00	24.00
5.00	4.00	3.00	5.00	4.00	5.00	26.00
5.00	5.00	**4.00**	4.00	3.00	5.00	26.00
5.00	5.00	5.00	4.00	5.00	5.00	29.00
5.00	4.00	5.00	4.00	5.00	5.00	28.00
4.00	3.00	5.00	4.00	4.00	4.00	24.00
4.00	3.00	5.00	5.00	5.00	3.00	25.00
4.00	4.00	5.00	*5.00*	5.00	5.00	28.00
5.00	5.00	4.00	5.00	5.00	4.00	28.00
3.00	4.00	5.00	3.00	5.00	4.00	24.00
5.00	5.00	5.00	5.00	5.00	5.00	30.00
5.00	5.00	5.00	5.00	5.00	5.00	30.00
5.00	5.00	5.00	5.00	5.00	5.00	30.00
5.00	5.00	5.00	4.00	5.00	5.00	29.00
1.00	1.00	4.00	1.00	3.00	3.00	13.00
4.00	5.00	4.00	4.00	5.00	5.00	27.00
5.00	5.00	5.00	5.00	5.00	5.00	30.00
4.00	5.00	5.00	4.00	5.00	5.00	28.00
4.00	5.00	4.00	4.00	5.00	4.00	26.00
4.00	5.00	4.00	4.00	5.00	4.00	26.00
4.00	5.00	5.00	4.00	5.00	4.00	27.00
4.00		5.00	4.00	4.00	5.00	22.00
4.00	3.00	5.00	3.00	5.00	5.00	25.00
4.00	2.00	5.00	3.00	5.00	5.00	24.00
5.00	5.00	5.00	4.00	3.00	5.00	27.00
4.00	5.00	5.00	4.00	5.00	5.00	28.00
5.00	5.00	5.00	5.00	4.00	5.00	29.00
5.00	5.00	5.00	3.00	4.00	4.00	26.00
4.00	5.00	5.00	3.00	5.00	5.00	27.00
4.00	3.00	5.00	5.00	4.00	4.00	25.00
5.00	5.00	**5.00**	4.00	5.00	5.00	29.00
**5.00**	4.00	5.00	5.00	5.00	5.00	29.00
5.00	5.00	4.00	5.00	5.00	5.00	29.00
5.00	5.00	5.00	3.00	5.00	5.00	28.00
4.00	5.00	4.00	4.00	5.00	4.00	26.00
5.00	5.00	4.00	4.00	5.00	4.00	27.00
5.00	*5.00*	4.00	4.00	5.00	4.00	27.00
4.00	5.00	5.00	4.00	3.00	4.00	25.00
5.00	3.00	3.00	4.00	4.00	5.00	24.00
4.00	**4.00**	5.00	4.00	5.00	4.00	26.00
5.00	5.00	5.00	5.00	5.00	5.00	30.00
4.00	5.00	5.00	4.00	5.00	4.00	27.00
4.00	3.00	4.00	4.00	3.00	5.00	23.00
4.00	5.00	5.00	4.00	5.00	5.00	28.00
4.00	5.00	5.00	4.00	5.00	5.00	28.00
4.00	5.00	5.00	3.00	5.00	5.00	27.00
4.00	3.00	5.00	3.00	5.00	4.00	24.00
4.00	5.00	4.00	4.00	5.00	**4.00**	26.00
4.00	4.00	5.00	5.00	5.00	5.00	28.00
5.00	5.00	5.00	3.00	5.00	5.00	28.00
4.00	4.00	3.00	5.00	5.00	4.00	25.00
4.00	4.00	3.00	5.00	5.00	4.00	25.00
4.00	4.00	5.00	4.00	5.00	5.00	27.00
4.00	4.00	5.00	*4.00*	5.00	5.00	27.00
4.00	4.00	5.00	4.00	5.00	5.00	27.00
4.00	3.00	5.00	5.00	5.00	4.00	26.00
5.00	4.00	5.00	4.00	5.00	5.00	28.00
4.00	5.00	5.00	4.00	5.00	5.00	28.00
5.00	5.00	4.00	5.00	4.00	3.00	26.00
5.00	4.00	4.00	5.00	4.00	3.00	25.00

5.00	5.00	5.00	4.00	5.00	4.00	28.00
5.00	5.00	5.00	4.00	5.00	4.00	28.00
5.00	5.00	5.00	4.00	5.00	4.00	28.00
4.00	5.00	5.00	4.00	2.00	5.00	25.00
5.00	5.00	5.00	3.00	5.00	5.00	28.00
4.00	5.00	4.00	4.00	5.00	5.00	27.00
4.00	5.00	4.00	4.00	5.00	5.00	27.00
5.00	5.00	4.00	4.00	5.00	4.00	27.00
5.00	5.00	5.00	4.00	3.00	3.00	25.00
4.00	5.00	5.00	5.00	5.00	5.00	29.00
4.00	5.00	5.00	5.00	5.00	5.00	29.00
4.00	5.00	5.00	4.00	5.00	5.00	28.00
4.00	5.00	5.00	5.00	5.00	4.00	28.00
5.00	5.00	5.00	3.00	5.00	4.00	27.00
3.00	5.00	5.00	4.00	3.00	5.00	25.00
4.00	5.00	5.00	4.00	5.00	4.00	27.00
5.00	5.00	5.00	4.00	3.00	4.00	26.00
5.00	5.00	5.00	4.00	3.00	4.00	26.00
5.00	**5.00**	5.00	4.00	3.00	4.00	26.00
5.00	5.00	5.00	4.00	3.00	4.00	26.00
4.00	5.00	4.00	4.00	5.00	4.00	26.00
4.00	3.00	5.00	3.00	5.00	4.00	24.00
4.00	5.00	5.00	3.00	4.00	5.00	26.00
4.00	5.00	5.00	3.00	4.00	5.00	26.00
5.00	5.00	5.00	4.00	5.00	5.00	29.00
4.00	5.00	5.00	4.00	4.00	5.00	27.00
5.00	4.00	5.00	4.00	5.00	4.00	27.00
4.00	5.00	4.00	3.00	5.00	5.00	26.00
3.00	4.00	4.00	5.00	5.00	4.00	25.00
4.00	3.00	5.00	3.00	4.00	4.00	23.00
5.00	5.00	5.00	4.00	5.00	5.00	29.00
5.00	5.00	5.00	4.00	5.00	5.00	29.00
5.00	5.00	5.00	3.00	5.00	4.00	27.00
4.00	5.00	5.00	4.00	5.00	4.00	27.00
5.00	5.00	5.00	4.00	5.00	4.00	28.00
5.00	5.00	5.00	4.00	5.00	4.00	28.00
4.00	5.00	4.00	4.00	5.00	5.00	27.00
4.00	5.00	5.00	4.00	5.00	4.00	27.00
4.00	5.00	5.00	3.00	5.00	3.00	25.00
5.00	4.00	5.00	5.00	5.00	4.00	28.00
4.00	5.00	5.00	2.00	5.00	5.00	26.00
4.00	5.00	5.00	2.00	5.00	5.00	26.00
4.00	5.00	5.00	3.00	4.00	4.00	25.00
4.00	5.00	5.00	3.00	4.00	4.00	25.00
4.00	5.00	5.00	4.00	5.00	5.00	28.00
5.00	5.00	4.00	5.00	5.00	5.00	29.00
5.00	5.00	5.00	4.00	5.00	4.00	28.00
5.00	4.00	3.00	5.00	5.00	5.00	27.00
4.00	5.00	4.00	4.00	5.00	5.00	27.00
4.00	5.00	4.00	4.00	5.00	5.00	27.00
4.00	4.00	5.00	4.00	5.00	5.00	27.00
4.00	4.00	5.00	4.00	5.00	5.00	27.00
4.00	4.00	5.00	4.00	5.00	5.00	27.00
5.00	4.00	5.00	3.00	3.00	5.00	25.00
4.00	4.00	4.00	5.00	4.00	4.00	25.00
2.00	4.00	5.00	2.00	5.00	5.00	23.00
2.00	4.00	5.00	2.00	5.00	5.00	23.00
2.00	4.00	5.00	2.00	5.00	5.00	23.00
2.00	4.00	5.00	2.00	5.00	5.00	23.00
2.00	4.00	5.00	2.00	5.00	5.00	23.00
2.00	4.00	5.00	2.00	5.00	5.00	23.00
2.00	4.00	5.00	2.00	5.00	5.00	23.00
2.00	4.00	5.00	2.00	5.00	5.00	23.00
1.00	5.00	5.00	4.00	5.00	5.00	25.00
5.00	4.00	5.00	3.00	5.00	5.00	27.00
5.00	4.00	5.00	3.00	5.00	5.00	27.00
5.00	4.00	5.00	3.00	5.00	5.00	27.00
5.00	4.00	5.00	3.00	5.00	5.00	27.00
3.00	3.00	5.00	5.00	5.00	4.00	25.00
3.00	5.00	5.00	4.00	5.00	4.00	26.00
4.00	4.00	5.00	4.00	1.00	5.00	23.00
5.00	5.00	5.00	4.00	4.00	5.00	28.00
4.00	5.00	5.00	4.00	5.00	5.00	28.00
4.00	5.00	5.00	4.00	5.00	5.00	28.00
4.00	5.00	5.00	3.00	5.00	5.00	27.00
4.00	5.00	5.00	5.00	4.00	4.00	27.00
4.00	5.00	5.00	4.00	5.00	4.00	27.00
4.00	5.00	5.00	4.00	5.00	4.00	27.00
3.00	2.00	4.00	4.00	4.00	4.00	21.00
5.00	5.00	4.00	4.00	5.00	5.00	28.00
5.00	4.00	5.00	4.00	5.00	4.00	27.00

4.00	5.00	5.00	4.00	5.00	5.00	28.00
3.00	5.00	5.00	3.00	4.00	3.00	23.00
3.00	3.00	4.00	4.00	4.00	5.00	23.00
5.00	5.00	4.00	4.00	5.00	5.00	28.00
5.00	5.00	5.00	3.00	5.00	4.00	27.00
3.00	5.00	4.00	4.00	4.00	5.00	25.00
4.00	5.00	5.00	4.00	5.00	5.00	28.00
4.00	3.00	5.00	5.00	5.00	5.00	27.00
5.00	3.00	5.00	4.00	5.00	5.00	27.00
4.00	4.00	5.00	3.00	4.00	**4.00**	24.00
4.00	5.00	5.00	3.00	5.00	5.00	27.00
5.00	5.00	5.00	**4.00**	5.00	4.00	28.00
5.00	5.00	5.00	3.00	5.00	5.00	28.00
5.00	5.00	**5.00**	3.00	5.00	4.00	27.00
5.00	4.00	4.00	4.00	5.00	2.00	24.00
4.00	5.00	5.00	4.00	5.00	4.00	27.00
5.00	5.00	5.00	4.00	4.00	5.00	28.00
4.00	4.00	3.00	4.00	5.00	5.00	25.00
4.00	4.00	5.00	4.00	4.00	4.00	25.00
4.00	5.00	4.00	4.00	1.00	2.00	20.00
3.00	5.00	5.00	5.00	4.00	4.00	26.00
5.00	4.00	4.00	3.00	2.00	2.00	20.00
5.00	4.00	5.00	5.00	5.00	4.00	28.00
5.00	5.00	5.00	5.00	5.00	5.00	30.00
5.00	4.00	5.00	4.00	5.00	4.00	27.00
4.00	4.00	5.00	4.00	5.00	5.00	27.00
4.00	4.00	5.00	4.00	5.00	5.00	27.00
4.00	4.00	5.00	4.00	5.00	5.00	27.00
4.00	4.00	5.00	4.00	5.00	5.00	27.00
4.00	4.00	5.00	4.00	5.00	4.00	26.00
4.00	5.00	5.00	5.00	5.00	5.00	29.00
4.00	3.00	5.00	3.00	4.00	4.00	23.00
5.00	5.00	5.00	4.00	4.00	3.00	26.00
5.00	5.00	5.00	3.00	4.00	3.00	25.00
5.00	5.00	5.00	3.00	4.00	3.00	25.00
4.00	3.00	5.00	4.00	4.00	5.00	25.00
**5.00**	5.00	5.00	5.00	5.00	5.00	30.00
5.00	4.00	5.00	3.00	5.00	5.00	27.00
5.00	4.00	5.00	3.00	5.00	5.00	27.00
3.00	1.00	3.00	2.00	5.00	1.00	15.00
4.00	5.00	5.00	5.00	5.00	5.00	29.00
4.00	4.00	5.00	5.00	5.00	5.00	28.00
4.00	5.00	5.00	4.00	5.00	5.00	28.00
5.00	3.00	5.00	5.00	5.00	5.00	28.00
2.00	3.00	4.00	4.00	5.00	4.00	22.00
4.00	5.00	4.00	5.00	5.00	4.00	27.00
4.00	4.00	5.00	4.00	4.00	5.00	26.00
5.00	4.00	3.00	4.00	3.00	4.00	23.00
5.00	5.00	5.00	5.00	5.00	5.00	30.00
4.00	5.00	5.00	4.00	5.00	5.00	28.00
5.00	5.00	5.00	4.00	4.00	4.00	27.00
5.00	4.00	5.00	3.00	5.00	3.00	25.00
5.00	3.00	5.00	3.00	5.00	3.00	24.00
5.00	4.00	4.00	3.00	5.00	4.00	25.00
4.00	5.00	5.00	4.00	5.00	4.00	27.00
5.00	5.00	5.00	4.00	5.00	5.00	29.00
5.00	5.00	5.00	4.00	5.00	5.00	29.00
5.00	5.00	5.00	4.00	5.00	5.00	29.00
5.00	4.00	5.00	5.00	5.00	5.00	29.00
4.00	5.00	4.00	4.00	5.00	5.00	27.00
5.00	5.00	5.00	5.00	5.00	4.00	29.00
5.00	4.00	5.00	3.00	4.00	5.00	26.00
5.00	5.00	5.00	5.00	4.00	4.00	28.00
5.00	5.00	4.00	4.00	5.00	4.00	27.00
5.00	4.00	4.00	4.00	5.00	4.00	26.00
5.00	4.00	5.00	3.00	5.00	4.00	26.00
4.00	4.00	5.00	3.00	5.00	4.00	25.00
4.00	4.00	5.00	5.00	4.00	4.00	26.00
4.00	5.00	5.00	3.00	4.00	5.00	26.00
4.00		5.00	5.00	4.00	4.00	22.00
5.00	4.00	5.00	4.00	4.00	4.00	26.00
4.00	5.00	5.00	3.00	5.00	4.00	26.00
4.00	5.00	5.00	4.00	5.00	4.00	27.00
4.00	4.00	5.00	5.00	4.00	4.00	26.00
4.00	3.00	5.00	4.00	4.00	4.00	24.00
5.00	4.00	5.00	5.00	5.00	5.00	29.00
3.00	4.00	5.00	4.00	4.00	5.00	25.00
4.00	5.00	5.00	5.00	5.00	4.00	28.00
5.00	5.00	5.00	4.00	4.00	5.00	28.00
5.00	4.00	3.00	5.00	5.00	5.00	27.00
5.00	5.00	5.00	5.00	5.00	5.00	30.00

**4.00**	4.00	5.00	3.00	4.00	3.00	23.00
4.00	5.00	5.00	3.00	5.00	**5.00**	27.00
4.00	5.00	5.00	3.00	5.00	5.00	27.00
4.00	3.00	5.00	4.00	3.00	4.00	23.00
4.00	3.00	5.00	4.00	3.00	3.00	22.00
4.00	5.00	5.00	4.00	5.00	5.00	28.00
5.00	5.00	5.00	4.00	5.00	5.00	29.00
4.00	**5.00**	5.00	4.00	4.00	2.00	24.00
5.00	4.00	5.00	4.00	5.00	5.00	28.00
5.00	4.00	5.00	5.00	4.00	4.00	27.00
5.00	4.00	5.00	4.00	5.00	4.00	27.00
4.00	4.00	5.00	3.00	2.00	2.00	20.00
4.00	4.00	5.00	3.00	2.00	2.00	20.00
4.00	4.00	5.00	3.00	2.00	2.00	20.00
4.00	5.00	5.00	5.00	5.00	5.00	29.00
**5.00**	5.00	4.00	4.00	4.00	5.00	27.00
3.00	5.00	5.00	4.00	5.00	4.00	26.00
5.00	3.00	5.00	3.00	4.00	4.00	24.00
4.00	4.00	4.00	3.00	4.00	3.00	22.00
4.00	4.00	4.00	2.00	4.00	3.00	21.00
4.00	5.00	5.00	5.00	5.00	4.00	28.00
5.00	5.00	5.00	5.00	5.00	5.00	30.00
5.00	5.00	5.00	5.00	5.00	5.00	30.00
5.00	5.00	5.00	5.00	5.00	5.00	30.00
5.00	5.00	5.00	5.00	5.00	5.00	30.00
3.00	5.00	5.00	4.00	5.00	4.00	26.00
3.00	5.00	5.00	4.00	5.00	4.00	26.00
5.00	5.00	5.00	3.00	4.00	4.00	26.00
5.00	5.00	5.00	4.00	5.00	5.00	29.00
5.00	5.00	5.00	4.00	5.00	5.00	29.00
3.00	5.00	5.00	4.00	4.00	5.00	26.00
3.00	5.00	5.00	4.00	4.00	5.00	26.00
5.00	5.00	5.00	4.00	4.00	3.00	26.00
4.00	4.00	5.00	4.00	5.00	4.00	26.00
4.00	3.00	5.00	4.00	5.00	4.00	25.00
4.00	3.00	5.00	4.00	5.00	4.00	25.00
4.00	3.00	5.00	4.00	5.00	4.00	25.00
4.00	3.00	5.00	4.00	5.00	4.00	25.00
4.00	4.00	5.00	3.00	5.00	4.00	25.00
3.00	4.00	5.00	4.00	4.00	3.00	23.00

**>15**	**>21**
1.00	1.00
1.00	1.00
1.00	1.00
1.00	1.00
1.00	1.00
1.00	1.00
1.00	1.00
1.00	1.00
1.00	1.00
1.00	1.00
1.00	1.00
1.00	1.00
1.00	1.00
1.00	1.00
1.00	1.00
1.00	1.00
0.00	0.00
1.00	1.00
1.00	1.00
1.00	1.00
1.00	1.00
1.00	1.00
1.00	1.00
1.00	1.00
1.00	1.00
1.00	1.00
1.00	1.00
1.00	1.00
1.00	1.00
1.00	1.00
1.00	1.00
1.00	1.00
1.00	1.00
1.00	1.00
1.00	1.00
1.00	1.00
1.00	1.00
1.00	1.00
1.00	1.00
1.00	1.00
1.00	1.00
1.00	1.00
1.00	1.00
1.00	1.00
1.00	1.00
1.00	1.00
1.00	1.00
1.00	1.00
1.00	1.00
1.00	1.00
1.00	1.00
1.00	1.00
1.00	1.00
1.00	1.00
1.00	1.00
1.00	1.00
1.00	1.00
1.00	1.00
1.00	1.00
1.00	1.00
1.00	1.00
1.00	1.00

1.00	1.00
1.00	1.00
1.00	1.00
1.00	1.00
1.00	1.00
1.00	1.00
1.00	1.00
1.00	1.00
1.00	1.00
1.00	1.00
1.00	1.00
1.00	1.00
1.00	1.00
1.00	1.00
1.00	1.00
1.00	1.00
1.00	1.00
1.00	1.00
1.00	1.00
1.00	1.00
1.00	1.00
1.00	1.00
1.00	1.00
1.00	1.00
1.00	1.00
1.00	1.00
1.00	1.00
1.00	1.00
1.00	1.00
1.00	1.00
1.00	1.00
1.00	1.00
1.00	1.00
1.00	1.00
1.00	1.00
1.00	1.00
1.00	1.00
1.00	1.00
1.00	1.00
1.00	1.00
1.00	1.00
1.00	1.00
1.00	1.00
1.00	1.00
1.00	1.00
1.00	1.00
1.00	1.00
1.00	1.00
1.00	1.00
1.00	1.00
1.00	1.00
1.00	1.00
1.00	1.00
1.00	1.00
1.00	1.00
1.00	1.00
1.00	1.00
1.00	1.00
1.00	1.00
1.00	1.00
1.00	1.00
1.00	1.00
1.00	1.00
1.00	1.00
1.00	1.00
1.00	1.00
1.00	1.00
1.00	1.00
1.00	1.00
1.00	1.00
1.00	1.00
1.00	1.00
1.00	1.00
1.00	1.00
1.00	1.00
1.00	1.00
1.00	1.00
1.00	1.00
1.00	1.00
1.00	1.00
1.00	1.00

1.00	1.00
1.00	1.00
1.00	1.00
1.00	1.00
1.00	1.00
1.00	1.00
1.00	1.00
1.00	1.00
1.00	1.00
1.00	1.00
1.00	1.00
1.00	1.00
1.00	1.00
1.00	1.00
1.00	1.00
1.00	1.00
1.00	1.00
1.00	1.00
1.00	1.00
1.00	0.00
1.00	1.00
1.00	0.00
1.00	1.00
1.00	1.00
1.00	1.00
1.00	1.00
1.00	1.00
1.00	1.00
1.00	1.00
1.00	1.00
1.00	1.00
1.00	1.00
1.00	1.00
1.00	1.00
1.00	1.00
1.00	1.00
1.00	1.00
1.00	1.00
1.00	1.00
1.00	0.00
1.00	1.00
1.00	1.00
1.00	1.00
1.00	1.00
1.00	1.00
1.00	1.00
1.00	1.00
1.00	1.00
1.00	1.00
1.00	1.00
1.00	1.00
1.00	1.00
1.00	1.00
1.00	1.00
1.00	1.00
1.00	1.00
1.00	1.00
1.00	1.00
1.00	1.00
1.00	1.00
1.00	1.00
1.00	1.00
1.00	1.00
1.00	1.00
1.00	1.00
1.00	1.00
1.00	1.00
1.00	1.00
1.00	1.00
1.00	1.00
1.00	1.00
1.00	1.00
1.00	1.00
1.00	1.00
1.00	1.00
1.00	1.00
1.00	1.00
1.00	1.00
1.00	1.00
1.00	1.00
1.00	1.00

1.00	1.00
1.00	1.00
1.00	1.00
1.00	1.00
1.00	1.00
1.00	1.00
1.00	1.00
1.00	1.00
1.00	1.00
1.00	1.00
1.00	1.00
1.00	0.00
1.00	0.00
1.00	0.00
1.00	1.00
1.00	1.00
1.00	1.00
1.00	1.00
1.00	1.00
1.00	1.00
1.00	1.00
1.00	1.00
1.00	1.00
1.00	1.00
1.00	1.00
1.00	1.00
1.00	1.00
1.00	1.00
1.00	1.00
1.00	1.00
1.00	1.00
1.00	1.00
1.00	1.00
1.00	1.00
1.00	1.00
1.00	1.00
1.00	1.00
1.00	1.00
1.00	1.00
1.00	1.00

**WHAT**	**affects**	**this**
Gender		Confidence in total
Year		Mx of crisis
Frequency to seeing patients		Score in total
Dental sch prep		confidence to dose adjust

Whether specific domain is weak

Confidence

	cardio	resp	neuro	bleeding	endo	cancer
1	9	7	23	9	46	34
2	17	32	47	30	56	80
3	62	68	94	76	82	87
4	135	124	86	122	63	50
5	41	33	14	27	17	13

Crisis

	cardio	resp	neuro	bleeding	endo	cancer
1	96	52	83	13	68	82
2	99	99	96	38	64	89
3	44	89	64	99	70	64
4	23	18	16	93	44	20
5	2	6	5	21	18	9

Score

	cardio	resp	neuro	bleeding	endo	cancer
1	2	2	0	1	37	2
2	9	3	2	17	23	6
3	19	30	11	54	21	28
4	125	84	51	135	58	95
5						
